# Health Care and Cybersecurity: Bibliometric Analysis of the Literature

**DOI:** 10.2196/12644

**Published:** 2019-02-15

**Authors:** Mohammad S Jalali, Sabina Razak, William Gordon, Eric Perakslis, Stuart Madnick

**Affiliations:** 1 MGH Institute for Technology Assessment Harvard Medical School Boston, MA United States; 2 Sloan School of Management Massachusetts Institute of Technology Cambridge, MA United States; 3 Division of General Internal Medicine Department of Medicine Brigham & Women’s Hospital Boston, MA United States; 4 Partners Healthcare Boston, MA United States; 5 Department of Dermatology Harvard Medical School Boston, MA United States; 6 Department of Biomedical Informatics Harvard Medical School Boston, MA United States

**Keywords:** bibliometric review, cybersecurity, health care, literature analysis, text mining

## Abstract

**Background:**

Over the past decade, clinical care has become globally dependent on information technology. The cybersecurity of health care information systems is now an essential component of safe, reliable, and effective health care delivery.

**Objective:**

The objective of this study was to provide an overview of the literature at the intersection of cybersecurity and health care delivery.

**Methods:**

A comprehensive search was conducted using PubMed and Web of Science for English-language peer-reviewed articles. We carried out chronological analysis, domain clustering analysis, and text analysis of the included articles to generate a high-level concept map composed of specific words and the connections between them.

**Results:**

Our final sample included 472 English-language journal articles. Our review results revealed that majority of the articles were focused on technology: Technology–focused articles made up more than half of all the clusters, whereas managerial articles accounted for only 32% of all clusters. This finding suggests that nontechnological variables (human–based and organizational aspects, strategy, and management) may be understudied. In addition, *Software Development Security*, *Business Continuity*, and *Disaster Recovery Planning* each accounted for 3% of the studied articles. Our results also showed that publications on *Physical Security* account for only 1% of the literature, and research in this area is lacking. Cyber vulnerabilities are not all digital; many physical threats contribute to breaches and potentially affect the physical safety of patients.

**Conclusions:**

Our results revealed an overall increase in research on cybersecurity and identified major gaps and opportunities for future work.

## Introduction

Cybersecurity is an increasingly critical aspect of health care information technology infrastructure. The rapid digitization of health care delivery, from electronic health records and telehealth to mobile health (mHealth) and network-enabled medical devices, introduces risks related to cybersecurity vulnerabilities [1]. These vulnerabilities are particularly worrisome because cyberattacks in a health care setting can result in the exposure of highly sensitive personal information or cause disruptions in clinical care [2-5]. Cyberattacks may also affect the safety of patients, for example, by compromising the integrity of data or impairing medical device functionality. The WannaCry and NotPetya ransomware attacks and vulnerabilities in Medtronic Implantable Cardiac Device Programmers are recent examples that have resulted in impaired health care delivery capabilities [6].

Health care organizations are particularly vulnerable to cyber threats. Verizon’s 2018 Data Breach Investigation Report found that the health care field, in general, was most affected by data breaches, which accounted for 24% of all investigated breaches across all industries [7]. Additionally, a report by the Ponemon Institute found that almost 90% of respondents (involved in health plans and health care clearing houses as well as health care providers with electronic health records) experienced a data breach in the past 2 years [8]. Another survey of health care information security professionals revealed that over 75% of health care organizations experienced a recent security incident [9]. The causes are multifactorial, involving both technology and people, and human error and cultural factors play increasingly critical roles [10,11]. Despite efforts to teach best-practice security behavior through training programs, recent surveys have revealed that one in five health care employees still write down their usernames and passwords on paper [12].

Given the increasing importance of cybersecurity for safe, effective, and reliable health care delivery, there is a need to provide an overview of the literature at the intersection of cybersecurity and health care. Recent systematic reviews synthesized insights from 31 articles on cyber threats in health care [13] and aggregated strategies from 13 articles about responding to cyber incidents in health care organizations [14]. In this study, we conducted a large bibliometric review of the literature and describe the current state of research on various aspects of cybersecurity in health care in order to not only understand current trends but also identify gaps and guide future research efforts toward improving the security of our health care systems.

## Methods

### Study Eligibility Criteria

A comprehensive search was conducted using PubMed and Web of Science (WoS) for English-language peer-reviewed articles. We identified search keywords by adopting terminologies in The National Initiative for Cybersecurity Careers and Studies [15] and The British Standards Institution glossaries [16]. The list of keywords used is as follows:

WoS (journal articles, all years):

“Health*” AND “Cybersecurity” OR “Cyber Security” OR “Cyber Attack*” OR “Cyber Crisis*” OR “Cyber Incident*” OR “Cyber Infrastructure*” OR “Cyber Operation*” OR “Cyber Risk*” OR “Cyber Threat*” OR “Cyberspace*” OR “Data Breach*” OR “Data Security*” OR “Firewall*” OR “Information Security*” OR “Information Systems Security*” OR “Information Technology Security*” OR “IT Security*” OR “Malware*” OR “Phishing*” OR “Ransomware*” OR “Security Incident*” OR “Information Assurance*”

PubMed (journal articles, all years, abstract availability):

“Cybersecurity” OR “Cyber Security” OR “Cyber Attack” OR “Cyber Crisis” OR “Cyber Incident” OR “Cyber Infrastructure” OR “Cyber Operation” OR “Cyber Risk” OR “Cyber Threat” OR “Cyberspace” OR “Data Breach” OR “Data Security” OR “Firewall” OR “Information Security” OR “Information Systems Security” OR “Information Technology Security” OR “IT Security” OR “Malware” OR “Phishing” OR “Ransomware” OR “Security Incident” OR “Information Assurance”.

Keywords that widened the search results far beyond the scope were rejected. For example, “exploit” and “malicious” can be used in a cyber context, but are more commonly used in unrelated contexts that add noise to the search. Such terms were not included because of their contribution to an overwhelming amount of irrelevant results.

We included articles published from the inception of PubMed in 1966 and WoS in 1900 to September 2017. Articles were excluded if they did not clearly focus on cybersecurity or health care or if they were reviews or meta-analyses. Inclusion and exclusion criteria were formulated prior to the preliminary title and abstract screening. The eligibility criteria were intentionally nonspecific to obtain a complete picture of the existing relevant research. To increase our confidence in the inclusion criteria, we conducted an initial pilot screening of 100 articles.

### Screening and Selection

Screening of titles and abstracts was conducted using the software package Abstrackr [17]. Full texts of the “maybe” articles were independently reviewed by two trained individuals to assess study eligibility. Disagreements about study inclusion were discussed until a consensus was reached. More details about our methodology are available in Multimedia Appendix 1.

### Chronological Clustering and Trend Analysis

We performed chronological analysis of the number of articles published per year and the number of authors per article. We topically clustered articles using 10 security domains created by the International Information Systems Security Certification Consortium to categorize each article (Multimedia Appendix 1). Each clustered article was further categorized as technological, managerial, legal, or interdisciplinary (if it fell into more than three categories). Features of the included articles, such as the publishing journal and number of citations, were recorded.

### Text Analysis

After analyzing all the titles and abstracts, we removed words with high frequencies that were common in research articles but were not specific to our subject (eg, “paper,” “using,” and “results”). In addition, we merged the plural forms with singular forms of the same word and merged “healthcare” and “health care” into “healthcare.” Subsequently, we created word clouds to visualize the word frequencies in titles and abstracts over time. Word frequency is represented by color and size, with darker, larger words representing higher occurrence.

We then assessed text titles and abstracts to generate a high-level concept map composed of specific words and the connections between them by using the software package Leximancer text analytics (version 4.5; Leximancer Pty Ltd, Brisbane, Australia). The software started with an unsupervised machine learning approach to extract a network of meaning from the data and developed a heat map that visually illustrated the end results. The method, underpinned by a naive Bayesian co-occurrence metric, considers how often two words co-occur as well as how often they occur apart [18,19]. Heat maps consist of “themes” represented by bubbles and “concepts” represented by grey dots. Concepts can be equated to a list of similar terms coalescing into a monothematic idea, and themes are clusters of these concepts. The lines between dots suggest a strong connection between two concepts.

## Results

### Search Results

**Figure 1:**
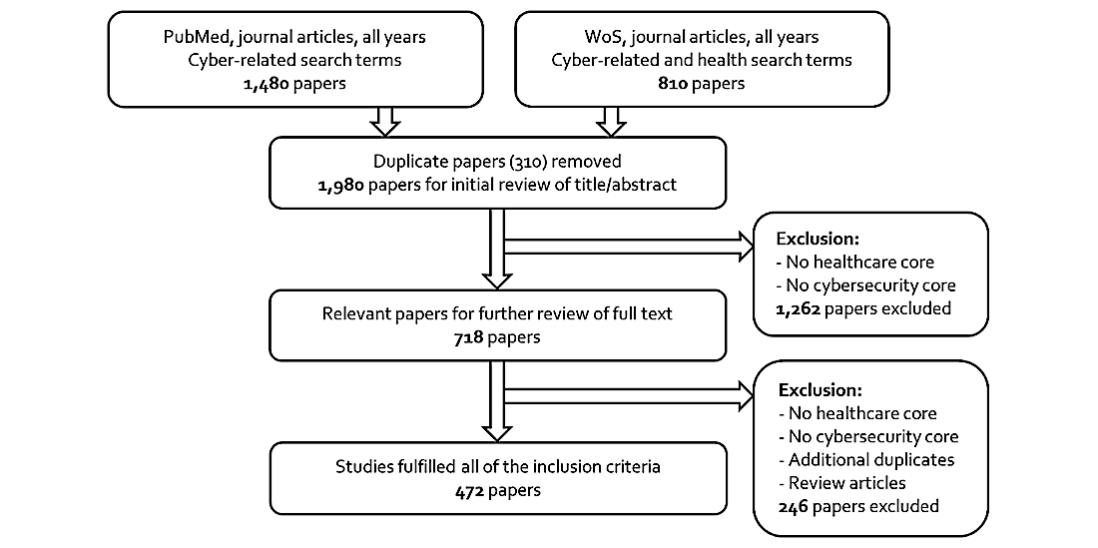
Search method and results.

The primary search on PubMed for papers containing terms pertaining to “cyber” yielded 1480 articles, and the search on WoS yielded 810 articles. After removing 310 duplicates, the titles and abstracts of 1980 articles were screened, which was facilitated by the Abstrackr software [17]. Based on the inclusion criteria, 1262 articles were excluded in the first screening, reducing the results to 718 articles for full-text review. Eventually, a further screening removed additional articles to provide a final selection of 472 articles. Figure 1 presents the search method and results.

### Chronological Clustering and Trend Analysis

**Figure 2:**
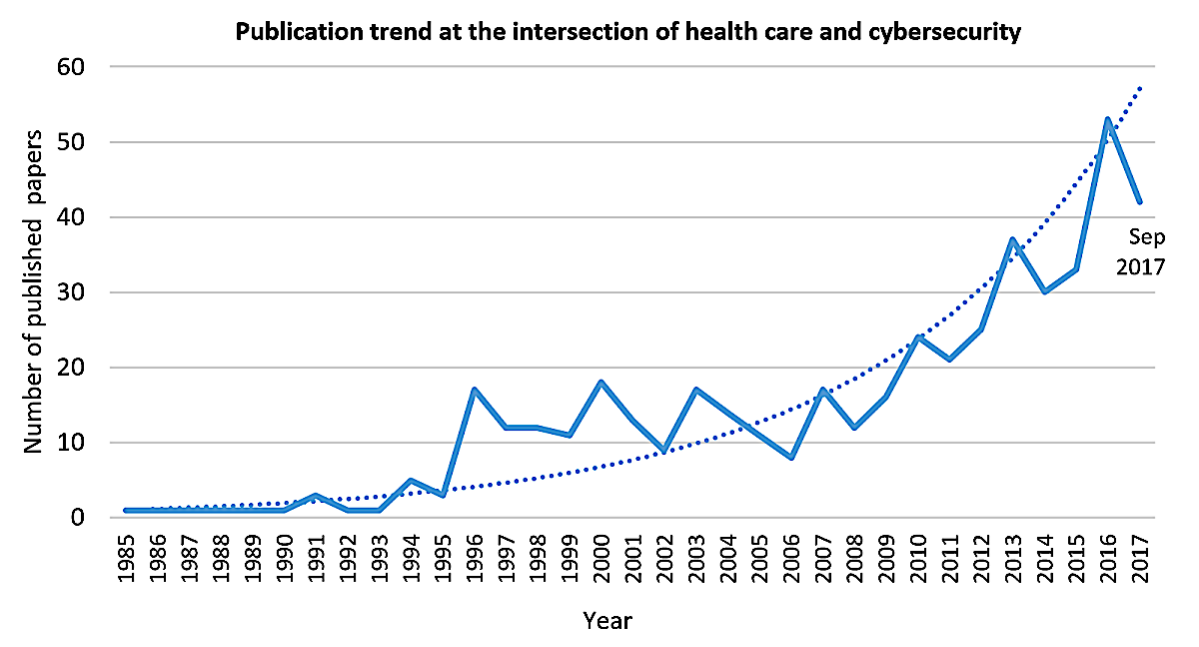
Annual number of published papers at the intersection of health care and cybersecurity (fitted trend line: y=0.9166e0.1252x; R²=0.82).

Figure 2 presents the overall trend of all publications over time, from 1985 to September 2017; the first included article was published in 1979 but was excluded from the figure for better visualization. Figure 2 shows a steady increase in the number of articles published on cybersecurity in health care (Multimedia Appendix 1).

**Figure 3:**
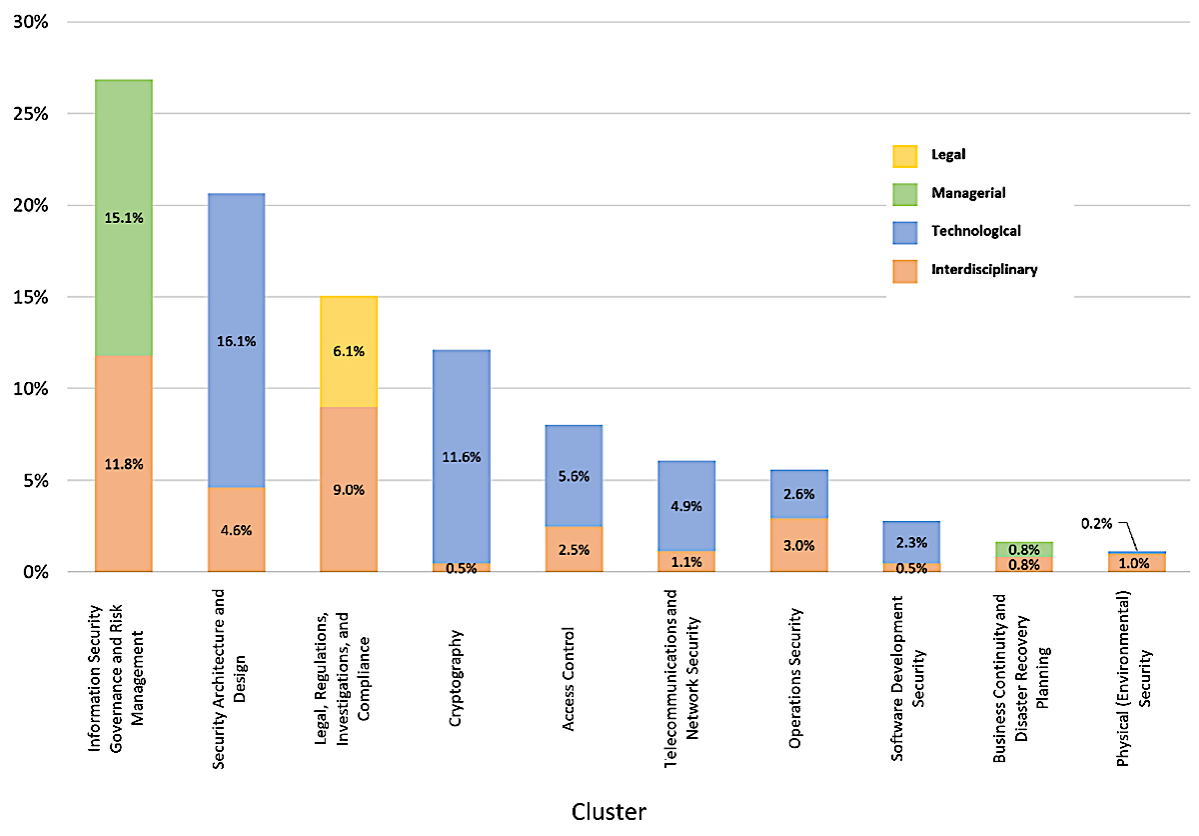
Cluster distributions.

Figure 3 shows the distribution among the three high-level categories: technological, managerial, and legal (Multimedia Appendix 1). The seven technological clusters made up more than half of all clusters, the two managerial clusters represented 32%, and the legal cluster represented 18% of all clusters.

The orange-shaded portion within each cluster in Figure 3 represents interdisciplinary articles (spanning multiple high-level categories). Although *Physical Security* had the lowest number of publications (Figure 3), it was the most interdisciplinary cluster (six out of the seven articles [85.7%] identified as interdisciplinary). *Legal, Regulations, Investigations, and Compliance* was the second most interdisciplinary cluster (59.8% of the articles in this category were interdisciplinary), followed by *Operations Security* (52.9%), *Business Continuity and Disaster Recovery Planning* (50%), *Information Security Governance and Risk Management* (43.9%), and *Access Control* (30.6%). Although *Security Architecture and Design* was the second most frequent cluster overall, only 22.2% of the articles were found to be interdisciplinary. The less interdisciplinary categories were *Telecommunications and Network Security* (18.9%), *Software Development Security* (17.6%), and *Cryptography* (4%) (Multimedia Appendix 1).

**Figure 4:**
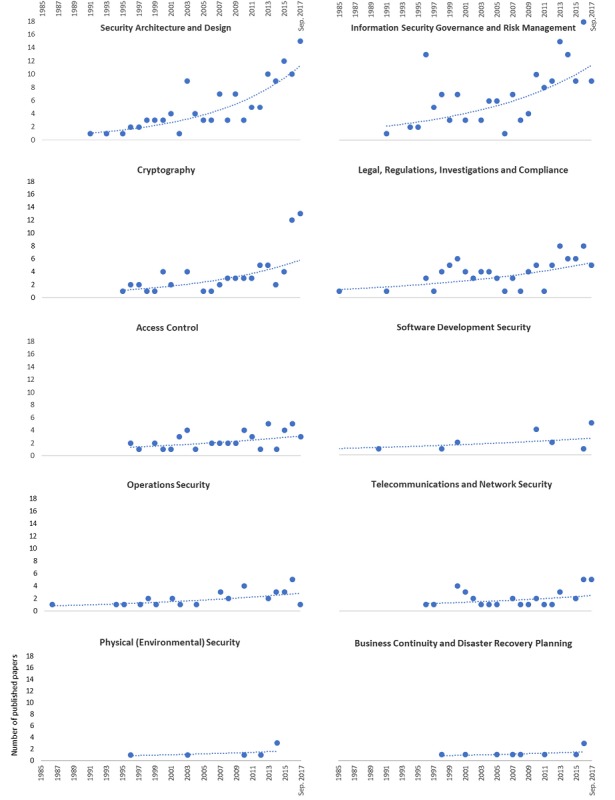
Trend of 10 clusters over time.

We analyzed the publication trends over time in the 10 clusters (Figure 4). All clusters showed increased frequency, and some clusters such as *Security Architecture and Design*, *Information Security Governance and Risk Management*, and *Cryptography* demonstrated particularly steep increases in frequency.

**Table 1:** Journals with the most articles.

Journal	Number of published papers	Indexed categories (according to Journal Citation Reports) [20]
Studies in Health Technology and Informatics	47	Not indexed
International Journal of Medical Informatics	24	Computer Science, Information Systems; Health Care Sciences & Services; Medical Informatics
Journal of Medical Systems	17	Health Care Sciences & Services; Medical Informatics
Journal of Diabetes Science and Technology	9	Not indexed
Healthcare Financial Management	8	Not indexed
Medical Informatics	8	Computer Science, Information Systems; Computer Science, Interdisciplinary Applications; Medical Informatics
International Journal of Bio-Medical Computing	8	Computer Science, Interdisciplinary Applications; Computer Science, Theory & Methods; Engineering, Biomedical; Medical Informatics
Computers & Security	7	Computer Science, Information Systems
Journal of the American Medical Informatics Association	7	Computer Science, Information Systems; Computer Science, Interdisciplinary Applications; Health Care Sciences & Services; Medical Informatics
Journal of Healthcare Protection Management	7	Not indexed
Telemedicine Journal and E-Health	5	Health Care Sciences & Services
IEEE^a^ Journal of Biomedical and Health Informatics	4	Computer Science, Information Systems; Computer Science, Interdisciplinary Applications; Mathematical & Computational Biology; Medical Informatics
Journal of the American Health Information Management Association	4	Not indexed
Journal of Digital Imaging	4	Radiology, Nuclear Medicine & Medical Imaging
Journal of Healthcare Information Management	4	Not indexed
Journal of Medical Internet Research	4	Health Care Sciences & Services; Medical Informatics
Journal of Medical Practice Management	4	Not indexed

^a^IEEE: Institute of Electrical and Electronics Engineers.

### Journal Characteristics

Overall, the 472 articles included were published in 239 unique journals. We ranked the journals according to the number of published articles and selected the journals with more than three articles, which resulted in a list of 17 journals (Table 1). According to the corresponding Incites Journal Citation Reports (JCR) categories [20], the top journals tended to focus on computer science, information systems, and medical informatics. The most popular JCR category, accounting for seven out of the 10 journals listed in JCR, was medical informatics. Six journals had a computer science category, specifically within information systems, interdisciplinary applications, or theory and methods. Five journals were from the health care sciences and services. Only one of the top 15 journals was categorized as a biomedical engineering journal; one, as a math and computational biology journal; and one, as a radiology, nuclear medicine, and medical imaging journal.

Approximately, 73% of the 239 journals had only published one article at the intersection of cybersecurity and health care. The high number and diversity of the journals included along with the low publication rate suggest that there is currently no major niche for medical practice readership at the intersection of cybersecurity and health care due to the cross-disciplinary nature of the field.

### Characteristics of the Most Cited Articles

Table 2 shows the most influential publications in the field of cybersecurity in health care, ranked by the number of citations as of September 2017. Six of the top 15 cited articles were published in five journals of the Institute of Electrical and Electronics Engineers. The clusters show a mix of article domains across the legal, managerial, and technological domains. The author-denoted keywords support this finding.

Of the total clusters of the top 15 articles, 38% belonged to *Security Architecture and Design*. *Cryptography* was the next most popular cluster (17%), followed by *Legal, Regulations, Investigations, and Compliance* (13%) and *Access Control* (13%). Overall, 79% of the clusters were technological, 13% were legal, and 8% were managerial. Additionally, 20% of the papers were interdisciplinary, with multiple clusters of distinct high-level categories. Notably, the list of most cited articles does not reflect the most recent articles, as citation of these articles is often significantly delayed.

**Table 2:** Top 15 most cited articles.

Rank	Number of citations	Title	Authors	Year	Journal	Clusters	Author-denoted keywords
1	443	Data security and privacy in wireless body area networks	Li M, Lou WJ, and Ren K	2010	IEEE^a^ Wireless Communications	Telecommunications and Network Security	Data security; Data privacy; Body sensor networks; Biomedical monitoring; Wireless sensor networks; Wearable sensors; Wireless communication; Medical services; Application software; Patient monitoring
2	304	Analyzing regulatory rules for privacy and security requirements	Breaux TD and Anton AI	2008	IEEE^a^ Transactions on Software Engineering	Legal, Regulations, Investigations and Compliance	Data security and privacy; Laws and regulations; Compliance; Accountability; Requirements engineering
3	173	Medical image security in a HIPAA^b^ mandated PACS^c^ environment	Cao F, Huang HK, and Zhou XQ	2003	Computerized Medical Imaging and Graphics	Legal, Regulations, Investigations and Compliance; Security Architecture and Design	Data encryption; Picture archiving and communication system security; Image integrity; Digital imaging and communication in medicine; Compliance; Health insurance portability and accountability act
4	168	SPOC: A Secure and Privacy-Preserving Opportunistic Computing Framework for Mobile-Healthcare Emergency	Lu RX, Lin XD, and Shen XM	2013	IEEE^a^ Transactions on Parallel and Distributed Systems	Access Control; Security Architecture and Design	Mobile-healthcare emergency; Opportunistic computing; User-centric privacy access control; PPSPC
5	158	Authenticity and integrity of digital mammography images	Zhou XQ, Huang HK, and Lou SL	2001	IEEE^a^ Transactions on Medical Imaging	Cryptography; Telecommunications and Network Security	Data embedding and cryptography; Digital mammography; Image authenticity and integrity; Telemammography
6	131	Security in health-care information systems--current trends	Smith E and Eloff JH	1999	International Journal of Medical Informatics	Access Control; Information Security Governance and Risk Management	Health-care information systems security; Risk-analysis in health-care information systems; Access control for computerized health-care; Electronic patient record; International Medical Informatics Association; Managed health-care
7	112	How to ensure data security of an epidemiological follow-up: quality assessment of an anonymous record linkage procedure	Quantin C, Bouzelat H, Allaert FA, Benhamiche AM, Faivre J, and Dusserre L	1998	International Journal of Medical Informatics	Cryptography; Security Architecture and Design	Data security; Computerized record; Linkage procedure
8	103	IBE-Lite: a lightweight identity-based cryptography for body sensor networks	Tan CC, Wang HD, Zhong S, and Li Q	2009	IEEE^a^ Transactions on Information Technology in Biomedicine	Security Architecture and Design; Cryptography	Body sensor network; Identity-based encryption; Privacy; Security
9	89	A security architecture for interconnecting health information systems	Gritzalis D and Lambrinoudakis C	2004	International Journal of Medical Informatics	Access Control; Security Architecture and Design	Information systems security; Computer security; Medical data security; Medical Data Protection; Electronic healthcare records; Role-based access control
10	85	Biometric methods for secure communications in body sensor networks: Resource-efficient key management and signal-level data scrambling	Bui FM and Hatzinakos D	2008	Eurasip Journal on Advances in Signal Processing	Security Architecture and Design; Cryptography	Not available
11	84	mHealth data security: the need for HIPAA^b^-compliant standardization	Luxton DD, Kayl RA, and Mishkind MC	2012	Telemedicine Journal and E-Health	Software Development Security; Legal, Regulations, Investigations and Compliance	Security; HIPAA^b^; Encryption; Telehealth; Mobile health
12	82	Analysis of the security and privacy requirements of cloud-based electronic health records systems	Rodrigues JJ, de la Torre I, Fernandez G, and Lopez-Coronado M	2013	Journal of Medical Internet Research	Security Architecture and Design	Cloud-computing; eHealth; Electronic health records (EHRs); Privacy; Security
13	82	Health care management and information systems security: awareness, training or education?	Katsikas SK	2000	International Journal of Medical Informatics	Information Security Governance and Risk Management	Health information systems; Information systems security; Health care management; Education; Training; Awareness
14	82	Securing m-healthcare social networks: challenges, countermeasures and future directions	Zhou J, Cao ZF, Dong XL, Lin XD, and Vasilakos AV	2013	IEEE^a^ Wireless Communications	Security Architecture and Design	Mobile communication; Social network services; Medical services; Mobile computing; Personal digital assistants; Privacy; Network security; Electronic medical records
15	80	Privacy and data security in E-health: requirements from the user's perspective	Wilkowska W and Ziefle M	2012	Health Informatics Journal	Security Architecture and Design	E-health; Gender; Medical assistive technologies; Privacy; Security

^a^IEEE: Institute of Electrical and Electronics Engineers.

^b^HIPAA: Health Insurance Portability and Accountability Act.

^c^PACS: picture archiving and communication system.

### Text Analysis

**Figure 5:**
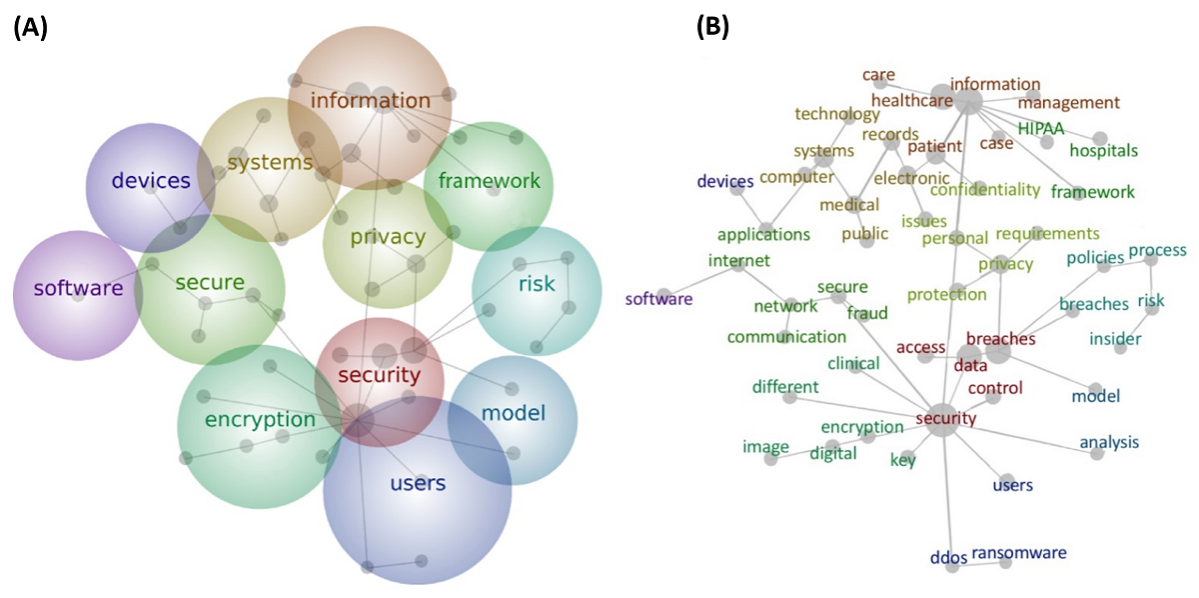
Thematic map of all titles and abstracts (A) and concept cloud of all titles and abstracts (B).

The text-mining analysis identified specific trends in the article texts. The map produced from all titles and abstracts is shown in Figure 5. The thematic bubbles are ranked by relevance based on a heat-map color scheme: Hot colors indicate more important themes, and cool colors indicate less important themes. The relative positions of the bubbles indicate the relationship between aggregated ideas, reflecting how closely they are related to each other. The sizes of the bubbles are only set to include their grey dots, and the size of each grey dot (a common word within the theme) indicates its relative frequency. The lines between these dots signify connectivity and association of concepts.

The overlay of grey-dot concepts onto thematic bubbles allows for more specific analysis of terms. Technological terms emerge as the main theme in Figure 5, including words like “encryption” and “software.” Concept words within these themes highlighted the following common elements of an organization’s informal technology structure related to cybersecurity: “Internet,” “network,” “applications,” “records,” “breaches,” “key,” and “electronic.” Managerial and legal terms were also identified as concepts (Figure 5). “Management” was a concept within the “information” theme. “Policies” and “process” were concepts in the risk theme and indicated the influence of risk analysis on the cybersecurity policies and procedures of organizations. “HIPAA” was a concept that stemmed from the “information” concept in the “important” theme.

The two central themes “security” and “information” included multiple, large grey-dot concepts that branched out into other thematic areas. There was an overlap between “security” and “encryption,” suggesting that encoding material is fundamental to security. An overlap between “security” and “users” could imply that user control is imperative to security.

For further analysis of word frequencies, the articles from 1985 to 2017 were split into four time periods: 1985-1993, 1994-2001, 2002-2009, and 2010-2017 (September). Multimedia Appendix 1 presents the word clouds within the four time periods. The size of the word represents the frequency of its occurrence. The term “privacy” increased in size in the last three time periods. “Internet” appeared in 1994-2001, around the time of the dot-com bubble. “Legal” was mentioned in 1985-1993, and “legislation” was found in 1994-2001. “HIPAA” appeared in 2002-2009 and again, although to a smaller extent, in 2010-2017.

**Figure 6:**
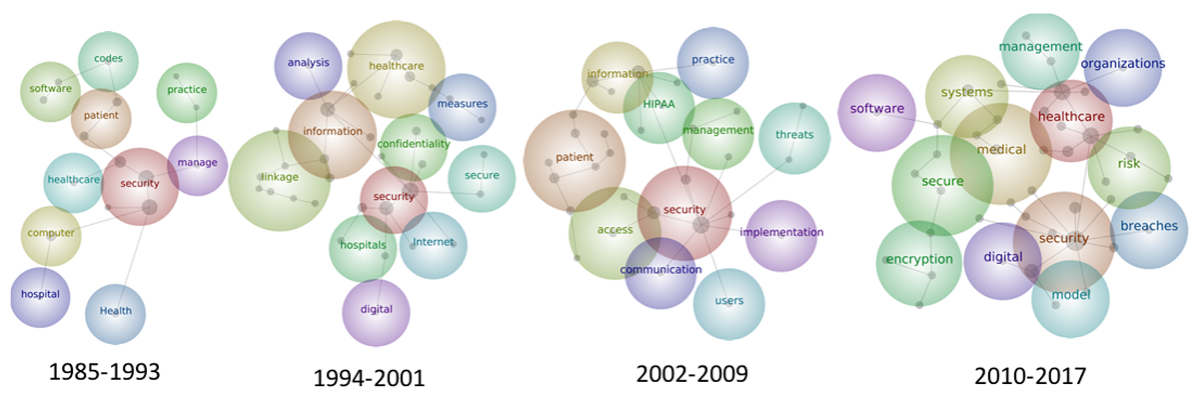
Thematic maps of titles and abstracts of articles in four time periods.

Maps of the four time periods were also created to identify trends over time (Figure 6). “Security” remained the most popular concept from 1985 to 2009, but was overtaken by “health care” from 2010 to 2017 (the most popular concept is indicated by the red bubble). The time period maps in Multimedia Appendix 1 provide further details.

## Discussion

### Overview

This article provides an analysis of the literature at the intersection of cybersecurity and health care. In general, research in this area has been increasing over the past 20 years and is continually represented in a wide, distributed array of academic journals, reflecting the importance of cybersecurity. With the increase in cybersecurity attacks against hospitals and dependency of health care delivery on technology, we expect cybersecurity to continue to play a central role in health care delivery.

Despite the increase in research and attention to cybersecurity, there are persistent shortcomings in the research on cybersecurity. For example, our research suggests that majority of the articles on cybersecurity focus on technology. In our domain-clustering analysis, technology–focused articles accounted for more than half of all the clusters, whereas managerial articles accounted for only 32%. Similarly, in our journal analysis, 58 articles included in the 15 most published journals were from computer science journals and 12 articles were from health-focused journals. Notably, 79% of the top 15 most cited paper clusters were technological. This focus on the technological aspects of cybersecurity suggests that nontechnological variables (human–based and organizational aspects, strategy, and management) may be understudied. Investment in technological tools should be the output of a robust cybersecurity strategy rather than the foundation [21]. An overwhelming majority of cybersecurity incidents are caused or propagated by people [22], and technological solutions can mitigate this risk to a limited extent.

We found discordance between the topics of the highly cited articles and the topical breakdown of our cluster analysis (these articles were published more than 5 years ago, implying that emergent threats are poorly captured). This finding suggests that articles on topics such as cryptography have significant traction, even though they are not widely present in the literature. On the other hand, only a few information security governance and compliance articles were frequently cited, despite accounting for a large portion of the literature.

Cybersecurity is most often examined with respect to privacy and compliance. Our results show that physical security is lacking in research, and only 1% of the literature is categorized under *Physical Security*. Not all cyber vulnerabilities are digital. Many physical threats contribute to breaches, and these threats potentially affect the physical safety of patients. *Software Development Security, Business Continuity*, and *Disaster Recovery Planning*, each accounted for 3% of the studied articles. Further examination is needed on these topics, and our study suggests that incident recovery (critical to the success of recovery from incidents) is not a significant focus in the research community. Articles focusing on legality were the least represented. Moreover, federal cybersecurity guidance such as the publications of the National Institute of Standards and Technology was seldom observed in our text analysis. In addition, massive increases in cybersecurity spending [23] did not drive proportional growth in the literature.

Our lexical analysis highlighted a separation of security processes and software terminology, with longer word distances between these themes. Additionally, the time period maps for 2002-2009 and 2010-2017 showed no overlap between the management and technological themes. More interdisciplinary research is needed to avoid gaps that arise from only analyzing managerial and technological security issues.

Unlike medical research, which is set up to openly benefit human lives [24], cybersecurity is based on the premise of an active adversary. The presence of this adversary may, unfortunately, drive a school of thought that knowledge, especially specific strategies and tactics, should not be shared openly, which impedes the growth and utility of research in this field.

### Limitations and Suggestions for Future Research

Our review was limited to journal articles indexed in PubMed and WoS. Information retrieval was limited to articles that included the terms of the search strategy in their titles or abstracts: Articles that used different terminology were not retrieved. Additionally, we only included articles with cybersecurity at the core of the study.

Our review did not assess non-English language articles or documents other than journal articles (eg, conference articles, white papers, or reports by governments or other organizations). A more comprehensive search could include these sources. Importantly, much of the work on cybersecurity and health care is operational and administrative, not academic. Information security professionals may not rely on academic literature as extensively as clinicians do when considering new diagnostics or therapeutics and may instead favor “on the job” experience and industry best practices. Additionally, information security research performed within the health care ecosystem may not be publishable due to security-related concerns such as exposing an internal vulnerability. Understanding the published literature in this space is an important starting point, and hospitals and patients will benefit from transparency in research, wherever possible.

Future reviews can focus on individual clusters that were reviewed in our study to provide a more in-depth analysis of the cluster. For instance, they could look specifically at business continuity and disaster recovery planning or software development security. Such a detailed focus can help synthesize research findings and provide best practices. Studies may also analyze the gap in managerial research and the implications of a narrow technological focus. Furthermore, such studies can focus on different settings in health care, such as inpatient and outpatient care, translational research, health and wellness environments, and integration of mobile devices and networked systems.

## Appendix

Multimedia Appendix 1 Details of the methodology.

## Abbreviations

HIPAA: Health Insurance Portability and Accountability Act
IEEE: Institute of Electrical and Electronics Engineers
mHealth: mobile health
NIST: National Institute of Standards and Technology
PACS: picture archiving and communication system
WoS: Web of Science

## References

[1] Jalali MS, Kaiser JP (2018). Cybersecurity in Hospitals: A Systematic, Organizational Perspective. J Med Internet Res.

[2] Gordon W, Fairhall A, Landman A (2017). Threats to Information Security - Public Health Implications. N Engl J Med.

[3] Perakslis E (2014). Cybersecurity in health care. N Engl J Med.

[4] Jarrett M (2017). Cybersecurity-A Serious Patient Care Concern. JAMA.

[5] Kramer D, Fu K (2017). Cybersecurity Concerns and Medical Devices: Lessons From a Pacemaker Advisory. JAMA.

[6] Furnell S, Emm D (2017). The ABC of ransomware protection. Computer Fraud & Security.

[7] (2018). Verizon Enterprise.

[8] (2016). Ponemon Institute.

[9] (2018). Healthcare Information and Management Systems Society.

[10] Madnick S, Jalali MS, Siegel M, Lee Y, Strong D, Wang R, Ang WH, Deng V, Mistree D, Woon W, Aung Z, Kramer O, Madnick S (2017). Measuring Stakeholders’ Perceptions of Cybersecurity for Renewable Energy Systems. Data Analytics for Renewable Energy Integration.

[11] Jalali MS, Siegel M, Madnick S (2018). Decision-making and biases in cybersecurity capability development: Evidence from a simulation game experiment. The Journal of Strategic Information Systems.

[12] Accenture (2018). Accenture 2018 Healthcare Workforce Survey on Cybersecurity.

[13] Kruse C, Frederick B, Jacobson T, Monticone DK (2017). Cybersecurity in healthcare: A systematic review of modern threats and trends. Technol Health Care.

[14] Jalali MS, Russell B, Razak S, Gordon WJ (2019). EARS to cyber incidents in health care. J Am Med Inform Assoc.

[15] (2017). National Initiative for Cybersecurity Careers and Studies.

[16] BSI.

[17] Wallace BC, Small K, Brodley CE, Lau J, Trikalinos TA (2012). Deploying an interactive machine learning system in an evidence-based practice center: abstrackr.

[18] Smith A, Humphreys MS (2006). Evaluation of unsupervised semantic mapping of natural language with Leximancer concept mapping. Behavior Research Methods.

[19] Cheng M, Edwards D (2017). A comparative automated content analysis approach on the review of the sharing economy discourse in tourism and hospitality. Current Issues in Tourism.

[20] (2018). Clarivate Analytics.

[21] (2017). Health Care Industry Cybersecurity Task Force: Report on improving cybersecurity in the health care industry.

[22] van Zadelhoff M (2016). Harvard Business Review.

[23] (2017). Gartner Forecasts Worldwide Security Spending Will Reach $96 Billion in 2018, Up 8 Percent from 2017.

[24] Ioannidis JPA, Greenland S, Hlatky MA, Khoury MJ, Macleod MR, Moher D, Schulz KF, Tibshirani R (2014). Increasing value and reducing waste in research design, conduct, and analysis. The Lancet.

