# Supplementary material for: Health Care and Cybersecurity: Bibliometric Analysis of the Literature
Source: J Med Internet Res. 2019 Feb 15;21(2):e12644. doi: 10.2196/12644 (PMC6396074; doi:10.2196/12644)
Supplement: Multimedia Appendix 1 [file jmir_v21i2e12644_app1.pdf]

Online supporting document

for

Health care and cybersecurity: a bibliometric analysis of the literature

Contents:

S1. Supplementary Notes: Details of the research method

S2. Supplementary Tables

S3. Supplementary Figures

S1. Supplementary Notes: Details of the research method

Screening and Selection

Search results were collated in Endnote and duplicates were deleted. The initial screening of titles and abstracts was conducted with Abstrackr [15] to mark each citation as 'include,' 'exclude,' or 'maybe.' Articles labeled 'exclude' and 'maybe' were tagged with an explanation for their irrelevance. Common tags were 'no/unclear cybersecurity core' and 'no/unclear health core.' Separate screenings were conducted for the WoS and PubMed articles to account for the differences in the databases. While health-relatedness was clearly identifiable in the PubMed titles/abstracts, we had to be more cautious when screening the WoS titles/abstracts.

Chronological, Clustering, and Trend Analysis

As discussed in the article, for clustering, we used ten security domains created by the International Information Systems Security Certification Consortium to categorize each article. The most widely known certification for information security professionals (Certified Information Systems Security Professional; CISSP) utilizes these ten clusters. See Table S1 in the Appendix for the list and definitions of the ten clusters.

The reviewers initially screened the title and abstract of each article to identify the most descriptive clusters. Multiple clusters per article were permitted. In the secondary screening, articles that were tagged 'full text' were further reviewed and categorized.

Characteristics of Most Publishing Journals and Most Cited Articles

In addition to collecting the features of publishing journals and the number of citations, we also grouped articles by journal and subject categories of the journals—drawn from Incites Journal Citation Reports (JCR) [19]. Moreover, we analyzed the most frequently cited articles. A list of the most cited articles was created by retrieving Google Scholar's article citation information (extracted in September 2017).

S2. Supplementary Tables

Table S1: List of Clusters (created by the International Information Systems Security Certification Consortium)

**Access Control**<br>"Access controls prevent unauthorized users from retrieving, using, or altering information. These controls are determined by an organization's risks, threats, and vulnerabilities."	Technological
**Telecommunications and Network Security**<br>"Network infrastructure, methods of communication, formats for transporting data, and measures taken to secure the network and transmission. The network is the vital link connecting information resources to users. Thus, this domain focuses on the design and architecture of the network and its components to prevent the disruption of data flow and intrusion."	
**Physical (Environmental) Security**<br>"The workplace environment and appropriate countermeasures used to physically protect information assets. Physical security includes access controls such as locks, guards, surveillance monitors, intrusion detectors, and alarms. It also includes appropriate control of computer equipment via a maintenance and inventory system, retention and storage, and a destruction process."	
**Operations Security**<br>"operations security is the actual process for implementing, maintaining, and monitoring safeguards and controls on a daily basis to prevent security incidents. Organizations can use numerous safeguards and controls to secure their operations."	
**Software Development Security**<br>"The software development security domain focuses on the systems development life cycle (SDLC) from system conception through its design, development, deployment, operation, and eventual retirement from service."	
**Cryptography**<br>"The cryptography domain concentrates on the methods of disguising information to ensure the integrity, confidentiality, and authenticity of information that is transmitted (i.e., data in transit) as well as information that is stored (i.e., data at rest). Cryptography ensures that both types of data are readable only by the appropriate, authorized individual."	
**Security Architecture and Design**<br>"Security architecture is fundamental to enforcing security policies that can be applied at different layers for each type of system platform. This architecture is based on how the enterprise will handle allowable and disallowable services and protocols, vulnerability scanning, patch management, firmware or software upgrades."	
**Business Continuity and Disaster Recovery Planning**<br>"Business continuity planning is the process of making the plans that will ensure that critical business functions can withstand a variety of emergencies. Disaster recovery planning involves making preparations for a disaster but also covers the procedures to be followed during and after a loss."	Managerial
**Information Security Governance and Risk Management**<br>"This domain identifies key security concepts, controls, and definitions. It also concentrates on many of the nontechnical aspects of information security while also addressing an analysis of technical risks."	
**Legal, Regulations, Investigations and Compliance**<br>"U.S. and international laws, regulations, and industry requirements pertaining to information security. This includes cybercrimes and the issues unique to investigating computer crimes. This domain also includes breach notification procedures."	Legal

Table S2: Publication Trend Counts

Year	Articles published
1985	1
1986	1
1987	1
1988	1
1989	1
1990	1
1991	3
1992	1
1993	1
1994	5
1995	3
1996	17
1997	12
1998	12
1999	11
2000	18
2001	13
2002	9
2003	17
2004	14
2005	11
2006	8
2007	17
2008	12
2009	16
2010	24
2011	21
2012	25
2013	37
2014	30
2015	33
2016	53
2017	42

Table S3: Cluster Distributions

Cluster	Legal	Managerial	Technological	Interdisciplinary	Total
Information Security Governance and Risk Management		15.1%		11.8%	26.9%
Security Architecture and Design			16.1%	4.6%	20.7%
Legal, Regulations, Investigations and Compliance	6.1%			9.0%	15.1%
Cryptography			11.6%	0.5%	12.1%
Access Control			5.6%	2.5%	8.0%
Telecommunications and Network Security			4.9%	1.2%	6.1%
Operations Security			2.6%	3.0%	5.6%
Software Development Security			2.3%	0.5%	2.8%
Business Continuity and Disaster Recovery Planning		0.8%		0.8%	1.6%
Physical (Environmental) Security			0.2%	1.0%	1.2%
**Total**					**%100**

Table S4: Article Clusters

Title	Author	Clusters
'Second generation' Internet e-health: the gladiator for HIPAA compliance?	Korpman, R. A. and J. S. Rose	Security Architecture and Design; Legal, Regulations, Investigations and Compliance
[A guide to good practice for information security in the handling of personal health data by health personnel in ambulatory care facilities]	Sanchez-Henarejos, A., J. L. Fernandez-Aleman, A. Toval, I. Hernandez-Hernandez, A. B. Sanchez-Garcia and J. M. C. d. Gea	Information Security Governance and Risk Management
[Clinical data protection and the internet]	Pommerening, K.	Telecommunications and Network Security; Cryptography
[Data security and the handling of patient data in home monitoring systems]	Heydenreich, F., C. Jurgens and F. Tost	Legal, Regulations, Investigations and Compliance
[Development of a secure and cost-effective infrastructure for the access of arbitrary web-based image distribution systems]	Hacklander, T., K. Kleber, H. Schneider, N. Demabre and B. M. Cramer	Security Architecture and Design; Operations Security
[E-health--challenge for health care system]	Buczak-Stec, E., K. Lemanowicz and M. Mazurek	Unclear*
[Information security in health care]	Kodmon, J. and Z. E. Csajbok	Legal, Regulations, Investigations and Compliance
[Measures to ensure data confidentiality in Clinical Units in order to achieve quality accreditation in Andalusia]	Carrasco Peralta, J. A., D. Nunez Garcia, M. M. Castellano-Zurera and A. T. Olivera	Unclear
[Medical data security in medico-legal opinioning]	Suslo, R. and B. Swiatek	Unclear
[Regulation of data protection in health care]	Kodmon, J.	Unclear
[Research on the security of medical image information and its related processing technologies]	Zhang, J. G., Zhongguo Yi, Liao Qi, Xie Za Zhi	Unclear
[Security aspects on the Internet]	Seibel, R. M., K. Kocher and P. Landsberg	Telecommunications and Network Security; Cryptography
[Security of healthcare data networks used for epidemiological studies]	Quantin, C., F. A. Allaert, H. Bouzelat, J. S. Rodrigues, B. Trombertpaviot, P. Brunet-Lecomte, F. Gremy and L. Dusserre	Software Development Security
[Security services: an overview of the French legislation on cryptography]	Quantin, C., E. Kerkri, F. A. Allaert, H. Bouzelat and L. Dusserre	Legal, Regulations, Investigations and Compliance; Cryptography
[Use of computers for patient data and billing]	Dilger, K.	Software Development Security
2008 HIMSS analytics report: security of patient data		Unclear
37% of hospitals perform cybersecurity incident response exercises annually		Information Security Governance and Risk Management; Business Continuity and Disaster Recovery Planning
A baseline security policy for distributed healthcare information systems	Gritzalis, D.	Information Security Governance and Risk Management
A Brief Chronology of Medical Device Security	Burns, A. J., M. E. Johnson and P. Honeyman	Unclear
A computerized record hash coding and linkage procedure to warrant epidemiological follow-up data security	Quantin, C., H. Bouzelat and L. Dusserre	Cryptography
A cybersecurity primer for translational research	Perakslis, E. D. and M. Stanley	Information Security Governance and Risk Management; Legal, Regulations, Investigations and Compliance
A data protection scheme for a remote vital signs monitoring healthcare service	Gritzalis, D. and C. Lambrinoudakis	Security Architecture and Design; Information Security Governance and Risk Management
A decision methodology for managing operational efficiency and information disclosure risk in healthcare processes	Bai, X., R. Gopal, M. Nunez and D. Zhdanov	Information Security Governance and Risk Management
A DNA-Based Encryption Method Based on Two Biological Axioms of DNA Chip and Polymerase Chain Reaction (PCR) Amplification Techniques	Zhang, Y., Z. Wang, Z. Wang, X. Liu and X. Yuan	Cryptography
A filter that prevents the spread of mail-attachment-type Trojan horse computer worms	Kobayashi, S., M. Goudge, T. Makie, E. Hanada, M. Harada and Y. Nose	Access Control
A Framework for an Effective Information Security Awareness Program in Healthcare A Case Study of Computer Game in Hospital Universiti Kebangsaan Malaysia	Ghazvini, A. and Z. Shukur	Information Security Governance and Risk Management
A Framework for Health Care Information Assurance Policy and Compliance	Cannoy, S. D. and A. F. Salam	Legal, Regulations, Investigations and Compliance; Information Security Governance and Risk Management
A generic methodology for health care data security	Furnell, S. M., P. N. Gaunt, G. Pangalos, P. W. Sanders and M. J. Warren	Unclear
A hybrid information security risk assessment procedure considering interdependences between controls	Lo, C. C. and W. J. Chen	Information Security Governance and Risk Management
A JAVA-based DICOM server with integration of clinical findings and DICOM-conform data encryption	Bernarding, J., A. Thiel and A. Grzesik	Cryptography
A Lightweight Encryption Scheme Combined with Trust Management for Privacy-Preserving in Body Sensor Networks	Guo, P., J. Wang, S. Ji, X. H. Geng and N. N. Xiong	Cryptography; Security Architecture and Design
A new approach to IT security	Kruger, D. and T. Anschutz	Security Architecture and Design
A new concept to ensure data privacy and data security in cancer registries	Michaelis, J., M. Miller, K. Pommerening and I. Schmidtmann	Cryptography; Security Architecture and Design

Title	Author	Clusters
A new method for generating an invariant iris private key based on the fuzzy vault system	Lee, Y. J., K. R. Park, S. J. Lee, K. Bae and J. Kim	Cryptography
A Novel Fuzzy Based Bio-Key Management scheme for Medical Data Security	Kalaivani, K. and R. Sivakumar	Cryptography; Telecommunications and Network Security
A proposed architecture and method of operation for improving the protection of privacy and confidentiality in disease registers	Churches, T.	Cryptography; Security Architecture and Design
A remote data access architecture for home-monitoring health-care applications	Lin, C. H., S. T. Young and T. S. Kuo	Security Architecture and Design
A secure and efficient Ciphertext-Policy Attribute-Based Signcryption for Personal Health Records sharing in cloud computing	Rao, Y. S.	Cryptography
A Secure Privacy-Preserving Data Aggregation Scheme Based on Bilinear ElGamal Cryptosystem for Remote Health Monitoring Systems	Ara, A., M. Al-Rodhaan, Y. Tian and A. Al-Dhelaan	Cryptography
A Secure Three-Factor User Authentication and Key Agreement Protocol for TMIS With User Anonymity	Amin, R. and G. P. Biswas	Security Architecture and Design; Cryptography
A security architecture for interconnecting health information systems	Gritzalis, D. and C. Lambrinoudakis	Access Control; Security Architecture and Design
A Socio-Technical Approach to Preventing, Mitigating, and Recovering from Ransomware Attacks	Sittig, D. F. and H. Singh	Operations Security; Information Security Governance and Risk Management; Business Continuity and Disaster Recovery Planning
A standardised graphic method for describing data privacy frameworks in primary care research using a flexible zone model	Kuchinke, W., C. Ohmann, R. A. Verheij, E. B. van Veen, T. N. Arvanitis, A. Taweel and B. C. Delaney	Security Architecture and Design
A study design to measure the outcomes of education in data security issues among health care professionals	Immonen, A., K. Saranto and K. Mauranen	Information Security Governance and Risk Management
A Study on Agent-Based Secure Scheme for Electronic Medical Record System	Chen, T. L., Y. F. Chung and F. Y. S. Lin	Access Control; Security Architecture and Design
A study on an information security system of a regional collaborative medical platform	Zhao, J., K. Peng, J. Leng, X. Sun, Z. Zhang, W. Xue and L. Ren	Information Security Governance and Risk Management
A study on user authentication methodology using numeric password and fingerprint biometric information	Ju, S. H., H. S. Seo, S. H. Han, J. C. Ryou and J. Kwak	Access Control
A tale of two standards: strengthening HIPAA security regulations using the PCI-DSS	Gaynor, M., C. Bass and B. Duepner	Legal, Regulations, Investigations and Compliance
A team approach to managing an information security program	Zakoworotny, C., C. Rutz and C. Zwingman-Bagley	Information Security Governance and Risk Management
Access control in healthcare: the methodology from legislation to practice	Ferreira, A., R. Correia, D. Chadwick and L. Antunes	Legal, Regulations, Investigations and Compliance; Access Control
Achieving Secure and Efficient Data Access Control for Cloud-Integrated Body Sensor Networks	Guan, Z. T., T. T. Yang and X. J. Du	Access Control; Cryptography
Addressing information security training and awareness within the European healthcare community	Furnell, S., P. Sanders and M. Warren	Information Security Governance and Risk Management
Advanced approach to information security management system model for industrial control system	Park, S. and K. Lee	Information Security Governance and Risk Management; Legal, Regulations, Investigations and Compliance
An agile enterprise regulation architecture for health information security management	Chen, Y. P., S. H. Hsieh, P. H. Cheng, T. N. Chien, H. S. Chen, J. J. Luh, J. S. Lai, F. Lai and S. J. Chen	Information Security Governance and Risk Management
An Efficient Cloud-Assisted Message Authentication Scheme in Wireless Body Area Network	Liu, H. J. and Y. H. Chen	Cryptography
An exploration of risk information search via a search engine: Queries and clicks in healthcare and information security	Wang, J. G., N. Xiao and H. R. Rao	Unclear
An information security management strategy for healthcare institutions	Grotan, T. O. and K. R. Iversen	Information Security Governance and Risk Management
Analysis of health professional security behaviors in a real clinical setting: an empirical study	Fernandez-Aleman, J. L., A. Sanchez-Henarejos, A. Toval, A. B. Sanchez-Garcia, I. Hernandez-Hernandez and L. Fernandez-Luque	Information Security Governance and Risk Management
Analysis of information security management systems at 5 domestic hospitals with more than 500 beds	Park, W. S., S. W. Seo, S. S. Son, M. J. Lee, S. H. Kim, E. M. Choi, J. E. Bang, Y. E. Kim and O. N. Kim	Information Security Governance and Risk Management
Analysis of the security and privacy requirements of cloud-based electronic health records systems	Rodrigues, J. J., I. de la Torre, G. Fernandez and M. Lopez-Coronado	Security Architecture and Design
Analyzing regulatory rules for privacy and security requirements	Breaux, T. D. and A. I. Anton	Legal, Regulations, Investigations and Compliance
Anonymity versus privacy: selective information sharing in online cancer communities	Frost, J., I. E. Vermeulen and N. Beekers	Unclear
Anti-malware software and medical devices		Software Development Security
Applying a Presentation Content Manifest for Signing Clinical Documents	Lien, C. Y., C. H. Hsiao, L. C. Huang and T. Kao	Cryptography
Architecture of authorization mechanism for medical data sharing on the grid	Tashiro, T., S. Date, S. Takeda, I. Hasegawa and S. Shimojo	Security Architecture and Design; Access Control

Title	Author	Clusters
Assessing and comparing information security in swiss hospitals	Landolt, S., J. Hirschel, T. Schlienger, W. Businger and A. M. Zbinden	Information Security Governance and Risk Management
Assessing staff attitudes towards information security in a European healthcare establishment	Furnell, S. M., P. N. Gaunt, R. F. Holben, P. W. Sanders, C. T. Stockel and M. J. Warren	Information Security Governance and Risk Management
Assessing the Security of Connected Diabetes Devices	Out, D. J. and O. Tettero	Security Architecture and Design
Assurance of energy efficiency and data security for ECG transmission in BASNs	Ma, T., P. L. Shrestha, M. Hempel, D. Peng, H. Sharif and H. H. Chen	Cryptography; Security Architecture and Design
Audited credential delegation: a usable security solution for the virtual physiological human toolkit	Haidar, A. N., S. J. Zasada, P. V. Coveney, A. E. Abdallah, B. Beckles and M. A. S. Jones	Security Architecture and Design
Authenticity and integrity of digital mammography images	Zhou, X. Q., H. K. Huang and S. L. Lou	Cryptography; Telecommunications and Network Security
Awareness Training Transfer and Information Security Content Development for Healthcare Industry	Ghazvini, A. and Z. Shukur	Information Security Governance and Risk Management
Beef up your information security with the new HIPAA-mandated standards		Legal, Regulations, Investigations and Compliance
Benchmarking HIPAA compliance	Wagner, J. R., D. J. Thoman, K. Anumalasetty, P. Hardre and T. Ross-Lazarov	Legal, Regulations, Investigations and Compliance
BENEFIT AND RISK ASSESSMENT OF COMPUTERIZED HEALTH CARDS - A CASE-STUDY	Nguyen, N. T., Y. Printz, S. Saadaoui and A. Nicolay	Security Architecture and Design
Biomedical Watermarking: An Emerging and Secure Tool for Data Security and Better Tele-Diagnosis in Modern Health Care System	Pal, K., G. Ghosh, M. Bhattacharya, R. Srivastava, S. K. Singh and K. K. Shukla	Security Architecture and Design
Biometric methods for secure communications in body sensor networks: Resource-efficient key management and signal-level data scrambling	Bui, F. M. and D. Hatzinakos	Security Architecture and Design; Cryptography
Brainjacking: Implant Security Issues in Invasive Neuromodulation	Pycroft, L., S. G. Boccard, S. L. Owen, J. F. Stein, J. J. Fitzgerald, A. L. Green and T. Z. Aziz	Security Architecture and Design
Breaching the security of the Kaiser Permanente Internet patient portal: the organizational foundations of information security	Collmann, J. and T. Cooper	Information Security Governance and Risk Management; Security Architecture and Design; Legal, Regulations, Investigations and Compliance
Business Model for the Security of a Large-Scale PACS, Compliance with ISO/27002:2013 Standard	Gutierrez-Martinez, J., M. A. Nunez-Gaona and H. Aguirre-Meneses	Information Security Governance and Risk Management; Legal, Regulations, Investigations and Compliance
Business process design. Securing computerized health information files	Barthel, C. W., C. M. Kalina and J. Fitko	Information Security Governance and Risk Management; Operations Security
Chief medical officer actions on information security in an Italian rehabilitation centre	Reni, G., M. Molteni, S. Arlotti and F. Pinciroli	Security Architecture and Design
Cloud-based privacy-preserving remote ECG monitoring and surveillance	Page, A., O. Kocabas, T. Soyata, M. Aktas and J. P. Couderc	Security Architecture and Design; Cryptography
CO-REGULATION OF ONLINE CONSUMER PERSONAL HEALTH RECORDS: BREAKING THROUGH THE PRIVACY LOGJAM TO INCREASE THE ADOPTION OF A LONG-OVERDUE TECHNOLOGY	Rank, P. S.	Legal, Regulations, Investigations and Compliance
Common object request broker architecture (CORBA)-based security services for the virtual radiology environment	Martinez, R., C. Cole, J. Rozenblit, J. F. Cook and A. K. Chacko	Security Architecture and Design
Compliance-Driven Architecture for Healthcare Industry	Gardazi, S. U. and A. A. Shahid	Software Development Security; Security Architecture and Design; Legal, Regulations, Investigations and Compliance
Concepts for a standard based cross-organisational information security management system in the context of a nationwide EHR	Mense, A., F. Hoheiser-Pfortner, M. Schmid and H. Wahl	Information Security Governance and Risk Management; Telecommunications and Network Security
Conceptual privacy framework for health information on wearable device	Safavi, S. and Z. Shukur	Security Architecture and Design
Conceptualizing the silent risk of inadvertent information leakages	Lechler, T. and S. Wetzel	Telecommunications and Network Security
Concern about security and privacy, and perceived control over collection and use of health information are related to withholding of health information from healthcare providers	Agaku, I. T., A. O. Adisa, O. A. Ayo-Yusuf and G. N. Connolly	Access Control
Confidential handling of data in secondary data research - Approaches to solving data concentration and data security problems	Ihle, P., J. Krappweis and I. Schubert	Security Architecture and Design; Operations Security
Confidentiality, data security, and cancer research: Perspectives from the National Cancer Institute		Legal, Regulations, Investigations and Compliance
Coordination or Collision? The Intersection of Diabetes Care, Cybersecurity, and Cloud-Based Computing	Thiel, S., J. Mitchell and J. Williams	Security Architecture and Design
CORBA security services for health information systems	Blobel, B. and M. Holena	Security Architecture and Design; Information Security Governance and Risk Management
Critical theory as an approach to the ethics of information security	Stahl, B. C., N. F. Doherty, M. Shaw and H. Janicke	Unclear

Title	Author	Clusters
Current medicolegal and confidentiality issues in large, multicenter research programs	Carney, P. A., B. M. Geller, H. Moffett, M. Ganger, M. Sewell, W. E. Barlow, N. Stalnaker, S. H. Taplin, C. Sisk, V. L. Ernster, H. A. Wilkie, B. Yankaskas, S. P. Poplack, N. Urban, M. M. West, R. D. Rosenberg, S. Michael, T. D. Mercurio and R. Ballard-Barbash	Legal, Regulations, Investigations and Compliance; Information Security Governance and Risk Management
Cyber crimes	Nuzback, K.	Unclear
Cyber risk and privacy liability: a click in the right direction?	McDonough, W. J.	Information Security Governance and Risk Management
Cyber-Security Issues in Healthcare Information Technology	Langer, S. G.	Legal, Regulations, Investigations and Compliance; Security Architecture and Design
Cybercare 2.0: meeting the challenge of the global burden of disease in 2030	Rosen, J. M., L. Kun, R. E. Mosher, E. Grigg, R. C. Merrell, C. Macedonia, J. Klaudt-Moreau, A. Price-Smith and J. Geiling	Telecommunications and Network Security
Cyberinfo: privacy and personal health data in cyberspace	Day, J.	Legal, Regulations, Investigations and Compliance; Security Architecture and Design
Cybersecurity and medical devices: A practical guide for cardiac electrophysiologists	Ransford, B., D. B. Kramer, D. Foo Kune, J. Auto de Medeiros, C. Yan, W. Xu, T. Crawford and K. Fu	Unclear
Cybersecurity and privacy issues for socially integrated mobile healthcare applications operating in a multi-cloud environment	Al-Muhtadi, J., B. Shahzad, K. Saleem, W. Jameel and M. A. Orgun	Security Architecture and Design
Cybersecurity and the Medical Device Product Development Lifecycle	Jones, R. W. and K. Katzis	Software Development Security
Cybersecurity for Connected Diabetes Devices	Klonoff, D. C.	Security Architecture and Design
Cybersecurity in Artificial Pancreas Experiments	O'Keeffe, D. T., S. Maraka, A. Basu, P. Keith-Hynes and Y. C. Kudva	Security Architecture and Design; Legal, Regulations, Investigations and Compliance
Cybersecurity in radiology: Access of public hot spots and public Wi-Fi and prevention of cybercrimes and HIPAA violations	Gerard, P., N. Kapadia, J. Acharya, P. T. Chang and Z. Lefkovitz	Telecommunications and Network Security
Cybersecurity in the Clinical Setting: Nurses' Role in the Expanding Internet of Things	Billingsley, L. a. S. A. M.	Information Security Governance and Risk Management
Cybersecurity Regulation of Wireless Devices for Performance and Assurance in the Age of Medjacking	Armstrong, D. G., D. N. Kleidermacher, D. C. Klonoff and M. J. Slepian	Information Security Governance and Risk Management; Security Architecture and Design
Cybersecurity: The heat is on	Morrissey, J.	Information Security Governance and Risk Management
Cyberterrorism: is the U.S. healthcare system safe?	Harries, D. and P. M. Yellowlees	Unclear
Danger is in the eye of the beholders: Social representations of Information Systems security in healthcare	Vaast, E.	Information Security Governance and Risk Management
Data damage assessment and recovery algorithm from malicious attacks in healthcare data sharing systems	Haraty, R. A., M. Zbib and M. Masud	Business Continuity and Disaster Recovery Planning
DATA LOSS PREVENTION AND CONTROL: INSIDE ACTIVITY INCIDENT MONITORING, IDENTIFICATION, AND TRACKING IN HEALTHCARE ENTERPRISE ENVIRONMENTS	Tu, M. H., K. Spoa-Harty and L. L. Xiao	Operations Security
Data protection and the clinician: guidelines on data security	Williamson, J. D.	Legal, Regulations, Investigations and Compliance
Data protection in grid-based multicentric clinical trials: killjoy or confidence-building measure?	Arning, M., N. Forgo and T. Krugel	Information Security Governance and Risk Management
Data security		Legal, Regulations, Investigations and Compliance
Data security and patient confidentiality: the manager's role	Fisher, F. a. B. M.	Information Security Governance and Risk Management; Legal, Regulations, Investigations and Compliance
Data Security and Privacy in Apps for Dementia: An Analysis of Existing Privacy Policies	Rosenfeld, L., J. Torous and I. V. Vahia	Unclear
DATA SECURITY AND PRIVACY IN WIRELESS BODY AREA NETWORKS	Li, M., W. J. Lou and K. Ren	Telecommunications and Network Security
Data security and protection in cross-institutional electronic patient records	van der Haak, M., A. C. Wolff, V. Mludek, P. Drings, M. Wannenmacher and T. Wetter	Legal, Regulations, Investigations and Compliance
Data security and protection in cross-institutional electronic patient records	van der Haak, M., A. C. Wolff, R. Brandner, P. Drings, M. Wannenmacher and T. Wetter	Security Architecture and Design
Data security assurance in CAD-PACS integration	Zhou, Z.	Cryptography; Security Architecture and Design
DATA SECURITY IN HEALTH INFORMATION-SYSTEMS BY APPLYING SOFTWARE TECHNIQUES	Sauter, K.	Software Development Security
Data security in medical computer systems	White, R.	Operations Security
Data security in medical information systems: technical aspects of a proposed legislation	Gritzalis, D., S. Katsikas, J. Keklikoglou and A. Tomaras	Legal, Regulations, Investigations and Compliance; Information Security Governance and Risk Management
Data security in occupational health	Damrongsak, M. and K. C. Brown	Information Security Governance and Risk Management; Access Control
Data security issues arising from integration of wireless access into healthcare networks	Frenzel, J. C.	Telecommunications and Network Security
Data security: an update		Operations Security; Access Control

Title	Author	Clusters
Data security: keeping a lid on Pandora's box		Security Architecture and Design; Operations Security
Deployment of a posteriori access control using IHE ATNA	Azkia, H., N. Cuppens-Boulahia, F. Cuppens, G. Coatrieux and S. Oulmakhzoune	Access Control
Design and develop a video conferencing framework for real-time telemedicine applications using secure group-based communication architecture	Mat Kiah, M. L., S. H. Al-Bakri, A. A. Zaidan, B. B. Zaidan and M. Hussain	Security Architecture and Design; Cryptography
Design of cloud security in the EHR for Indian healthcare services	Deshmukh, P.	Cryptography; Access Control
Design of cryptographically secure AES like S-Box using second-order reversible cellular automata for wireless body area network applications	Gangadari, B. R. and S. R. Ahamed	Cryptography
Design of Hack-Resistant Diabetes Devices and Disclosure of Their Cyber Safety	Sackner-Bernstein, J.	Security Architecture and Design
Design of secure access control scheme for personal health record-based cloud healthcare service	Liu, C. H., F. Q. Lin, C. S. Chen and T. S. Chen	Access Control; Security Architecture and Design
Determining the privacy policy deficiencies of health ICT applications through semi-formal modelling	Croll, P. R.	Information Security Governance and Risk Management
Determining the right level for your IT security investment	Claunch, D. and M. McMillan	Information Security Governance and Risk Management
Developing a Security Metrics Scorecard for Healthcare Organizations	Elrefaey, H., E. Borycki and A. Kushniruk	Information Security Governance and Risk Management
Developing the security culture at the SEISMED Reference Centres	Fowler, J.	Information Security Governance and Risk Management; Access Control; Physical (Environmental) Security
Development of a HIPAA-compliant environment for translational research data and analytics	Bradford, W., J. F. Hurdle, B. LaSalle and J. C. Facelli	Information Security Governance and Risk Management; Security Architecture and Design
Development of an E-Healthcare Information Security Risk Assessment Method	Wei, J. N., B. S. Lin and M. Loho-Noya	Information Security Governance and Risk Management
Development of information security baselines for healthcare information systems in New Zealand	Janczewski, L. and F. X. L. Shi	Legal, Regulations, Investigations and Compliance
Development of security guidelines for existing healthcare systems	Furnell, S. M., P. W. Sanders and M. J. Warren	Information Security Governance and Risk Management; Operations Security
Developments in health care, the increasing role of information technology: security issues	Immonen, S.	Information Security Governance and Risk Management
Digital Photograph Security: What Plastic Surgeons Need to Know	Thomas, V. A., P. B. Rugeley and F. H. Lau	Security Architecture and Design; Legal, Regulations, Investigations and Compliance
Digital signatures and the electronic health records: Providing legal and security guarantees	Bos, J. J.	Unclear
DNA-based cryptographic methods for data hiding in DNA media	Marwan, S., A. Shawish and K. Nagaty	Cryptography
Does the PCEHR mean a new paradigm for information security? Implications for health information management	Williams, P. A. H.	Legal, Regulations, Investigations and Compliance; Operations Security
Dual-channel in-line digital holographic double random phase encryption	Das, B., C. S. Yelleswarapu and D. V. Rao	Cryptography
Dual-Level Security based Cyclic18 Steganographic Method and its Application for Secure Transmission of Keyframes during Wireless Capsule Endoscopy	Muhammad, K., M. Sajjad and S. W. Baik	Cryptography
Early clinical experience with CardioCard - a credit card-sized electronic patient record	Bernheim, A. M., B. A. Schaer, C. Kaufmann, H. Brunner-La Rocca, N. Moulay-Lakhdar, P. T. Buser, M. E. Pfisterer and S. Osswald	Security Architecture and Design
ECG Data Encryption Then Compression Using Singular Value Decomposition	Liu, T. Y., K. J. Lin and H. C. Wu	Cryptography; Security Architecture and Design
Effective audit trails - A taxonomy for determination of information requirements	Asaro, P. V., R. L. Herting, A. C. Roth and M. R. Barnes	Access Control; Operations Security
Efficient and Privacy-Preserving Online Medical Prediagnosis Framework Using Nonlinear SVM	Zhu, H., X. Liu, R. Lu and H. Li	Cryptography; Security Architecture and Design
EMR confidentiality and information security	Kurtz, G.	Unclear
Enabling Location Privacy and Medical Data Encryption in Patient Telemonitoring Systems	Maglogiannis, I., L. Kazatzopoulos, K. Delakouridis and S. Hadjiefthymiades	Security Architecture and Design; Cryptography
Enhancing security and improving interoperability in healthcare information systems	Gritzalis, D. A.	Information Security Governance and Risk Management; Legal, Regulations, Investigations and Compliance
ENLIGHTENED REGULATORY CAPTURE	Thaw, D.	Legal, Regulations, Investigations and Compliance
Enriching healthcare applications with cryptographic mechanisms and XML- based security services	Bourka, A., A. Kaliontzoglou, D. Polemi, A. Georgoulas and P. Sklavos	Cryptography; Security Architecture and Design
eRegistries: governance for electronic maternal and child health registries	Myhre, S. L., J. Kaye, L. A. Bygrave, M. Aanestad, B. Ghanem, P. Mechael and J. F. Froen	Legal, Regulations, Investigations and Compliance
Ethical considerations for informed consent in infertility research: The use of electronic health records	Wells, K. J., J. R. Gordon, H. I. Su, S. Plosker and G. P. Quinn	Unclear
Ethical, legal and social issues for personal health records and applications	Cushman, R., A. M. Froomkin, A. Cava, P. Abril and K. W. Goodman	Information Security Governance and Risk Management

Title	Author	Clusters
Ethics, Privacy, and the Future of Genetic Information in Healthcare Information Assurance and Security	Springer, J. A., J. Beever, N. Morar, J. E. Sprague, M. D. Kane and A. I. R. Management	Unclear
Evaluating integration approaches adopted by healthcare organizations	Khoumbati, K. and M. Themistocleous	Unclear
Evaluation of a Cyber Security System for Hospital Network	Faysel, M. A.	Telecommunications and Network Security
Evaluation of existing district health management information systems a case study of the district health systems in Kenya	Odhiambo-Otieno, G. W.	Security Architecture and Design
Evaluation of Secure Computation in a Distributed Healthcare Setting	Kimura, E., K. Hamada, R. Kikuchi, K. Chida, K. Okamoto, S. Manabe, T. Kuroda, Y. Matsumura, T. Takeda and N. Mihara	Security Architecture and Design
Everday risk. Protecting against breach in release of information	McDavid, J. and R. Bowen	Information Security Governance and Risk Management
Experimental study on optical image encryption with asymmetric double random phase and computer-generated hologram	Xi, S., X. Wang, L. Song, Z. Zhu, B. Zhu, S. Huang, N. Yu and H. Wang	Cryptography
Explaining Users' Security Behaviors with the Security Belief Model	Williams, C. K., D. Wynn, R. Madupalli, E. Karahanna and B. K. Duncan	Information Security Governance and Risk Management
Exploring a New Security Framework for Remote Patient Monitoring Devices	Ondiege, B., M. Clarke and G. Mapp	Security Architecture and Design
Exploring the Far Side of Mobile Health: Information Security and Privacy of Mobile Health Apps on iOS and Android	Dehling, T., F. Gao, S. Schneider and A. Sunyaev	Security Architecture and Design
Extraction and anonymity protocol of medical file	Bouzelat, H., C. Quantin and L. Dusserre	Information Security Governance and Risk Management
Field test of classical symmetric encryption with continuous variables quantum key distribution	Jouguet, P., S. Kunz-Jacques, T. Debuisschert, S. Fossier, E. Diamanti, R. Alleaume, R. Tualle-Brouri, P. Grangier, A. Leverrier, P. Pache and P. Painchault	Cryptography
FINDING THE BEST OF THE IMPERFECT ALTERNATIVES FOR PRIVACY, HEALTH IT, AND CYBERSECURITY	Swire, P.	Legal, Regulations, Investigations and Compliance
Fingerprint verification on medical image reporting system	Chen, Y. C., L. K. Chen, M. D. Tsai, H. C. Chiu, J. S. Chiu and C. F. Chong	Security Architecture and Design; Access Control
Framework for securing personal health data in clinical decision support systems	Sandell, P.	Security Architecture and Design
From tangled webs to network integrity: computer security in hospital management. Part II	Ravella, J. A. and J. L. Gravel	Unclear
Fuzzy Assessment of Health Information System Users' Security Awareness	Aydin, O. M. and O. Chouseinoglou	Information Security Governance and Risk Management
Generating unique IDs from patient identification data using security models	Mohammed, E. A., J. C. Slack and C. T. Naugler	Security Architecture and Design; Cryptography
Genomic cloud computing: legal and ethical points to consider	Dove, E. S., Y. Joly, A. M. Tasse and B. M. Knoppers	Security Architecture and Design; Legal, Regulations, Investigations and Compliance
Genomics and privacy: implications of the new reality of closed data for the field	Greenbaum, D., A. Sboner, X. J. Mu and M. Gerstein	Security Architecture and Design; Information Security Governance and Risk Management
Grounding information security in healthcare	Ferreira, A., L. Antunes, D. Chadwick and R. Correia	Information Security Governance and Risk Management
Guidelines for computer security in general practice	Schattner, P., C. Pleteshner, H. Bhend and J. Brouns	Information Security Governance and Risk Management; Operations Security; Access Control
Health care data security: one size does not fit all	Krohn, R.	Unclear
Health care management and information systems security: awareness, training or education?	Katsikas, S. K.	Information Security Governance and Risk Management
Health care professionals protocol for secure online transmission of patient data	Goetz, C. F.	Security Architecture and Design; Legal, Regulations, Investigations and Compliance
Health care, an easy target, needs to get its guard up	Ladika, S.	Information Security Governance and Risk Management
Health Data Security Issues	Mantas, J. and J. Liaskos	Access Control
Health data security: a new priority	Siwicki, B.	Unclear
Health informatics: an intercultural perspective	Le, Q.	Unclear
Health informatics: handle with caution	Salamon, R., V. Leroy, S. Maurice-Tison and B. L. Blanc	Unclear
Health Information Security in Hospitals: the Application of Security Safeguards	Mehraeen, E., H. Ayatollahi and M. Ahmadi	Information Security Governance and Risk Management
Health information security: a case study of three selected medical centers in iran	Hajrahimi, N., S. M. Dehaghani and A. Sheikhtaheri	Information Security Governance and Risk Management; Access Control
Health IT for Patient Safety and Improving the Safety of Health IT	Magrabi, F., M. S. Ong and E. Coiera	Software Development Security
Health-Care Data Protection and Biometric Authentication Policies: Comparative Culture and Technology Acceptance in China and in the United States	Brown, C. L.	Information Security Governance and Risk Management

Title	Author	Clusters
Health-Care Security Strategies for Data Protection and Regulatory Compliance	Kwon, J. and M. E. Johnson	Operations Security
Healthcare information security. The threats and the safeguards--and how to manage them		Information Security Governance and Risk Management; Telecommunications and Network Security; Legal, Regulations, Investigations and Compliance; Operations Security
Healthcare IT trends raise bar for information security	Glaser, J. and J. Aske	Information Security Governance and Risk Management; Operations Security
Healthcare SaaS Based on a Data Model with Built-In Security and Privacy	Asija, R. and R. Nallusamy	Security Architecture and Design
Healthcare teams over the Internet: programming a certificate-based approach	Georgiadis, C. K., I. K. Mavridis and G. I. Pangalos	Access Control; Security Architecture and Design
Hierarchical data security in a Query-By-Example interface for a shared database	Taylor, M.	Security Architecture and Design; Access Control
High level security policies for health: from theory to practice	Defteraios, S., C. Lambrinoudakis and D. Gritzalis	Unclear
HIPAA for cancer educators: are you correctly using PHI?	Searson, S., J. Hicks, J. Cole, T. Herzig and C. M. Brooks	Legal, Regulations, Investigations and Compliance
HIPAA security standards: is your facility ready?		Legal, Regulations, Investigations and Compliance
HIPAA's impact on healthcare	Hellerstein, D.	Legal, Regulations, Investigations and Compliance
Hospital looks for data security, also gets improved productivity		Unclear
How secure is the Internet for healthcare applications?	Campbell, L. A.	Security Architecture and Design
How secure is your information system? An investigation into actual healthcare worker password practices	Cazier, J. A. and B. D. Medlin	Access Control
How Secure Is Your Radiology Department? Mapping Digital Radiology Adoption and Security Worldwide	Stites, M. and O. S. Pianykh	Access Control; Operations Security
How to ensure data security of an epidemiological follow-up: quality assessment of an anonymous record linkage procedure	Quantin, C., H. Bouzelat, F. A. Allaert, A. M. Benhamiche, J. Faivre and L. Dusserre	Cryptography; Security Architecture and Design
IBE-Lite: A Lightweight Identity-Based Cryptography for Body Sensor Networks	Tan, C. C., H. D. Wang, S. Zhong and Q. Li	Security Architecture and Design; Cryptography
Identification of Measures and Indicators for the IT Security of Networked Medical Devices: A Delphi Study	Leber, S. and E. Ammenwerth	Telecommunications and Network Security; Information Security Governance and Risk Management
Impact of HIPAA provisions on the stock market value of healthcare institutions, and information security and other information technology firms	Khansa, L., D. F. Cook, T. James and O. Bruyaka	Legal, Regulations, Investigations and Compliance
Implementation of data security and data privacy provisions will bring sweeping changes to laboratory service providers	Boothe, J. F.	Legal, Regulations, Investigations and Compliance
Implementation of information security and confidentiality policies, procedures, and standards	Perry, J. W.	Information Security Governance and Risk Management
Implementation of Medical Information Exchange System Based on EHR Standard	Han, S. H., M. H. Lee, S. G. Kim, J. Y. Jeong, B. N. Lee, M. S. Choi, I. K. Kim, W. S. Park, K. Ha, E. Cho, Y. Kim and J. B. Bae	Security Architecture and Design
Implementing Context and Team Based Access Control in healthcare intranets	Georgiadis, C. K., I. K. Mavridis, G. Nikolakopoulou and G. I. Pangalos	Operations Security; Access Control
Implementing data privacy and security (the Slovenian experience)	Markota, M. and G. Raic	Legal, Regulations, Investigations and Compliance
Implementing healthcare information security: standards can help	Orel, A. and I. Bernik	Information Security Governance and Risk Management; Legal, Regulations, Investigations and Compliance
Implementing technological safeguards to ensure patient privacy	Leestma, R.	Legal, Regulations, Investigations and Compliance
Improving computer security by health smart card	Nisand, G., F. A. Allaert, R. Brezillon, W. Isphording and N. Roeslin	Security Architecture and Design
Improving Individual Acceptance of Health Clouds through Confidentiality Assurance	Ermakova, T., B. Fabian and R. Zarnekow	Information Security Governance and Risk Management
Improving outcomes with interoperable EHRs and secure global health information infrastructure	Kun, L., G. Coatrieux, C. Quantin, R. Beuscart and R. Mathews	Unclear
Improving the redistribution of the security lessons in healthcare: An evaluation of the Generic Security Template	He, Y. and C. Johnson	Information Security Governance and Risk Management
Including Internet insurance as part of a hospital computer network security plan	Riccardi, K.	Telecommunications and Network Security
Incremental adoption of information security in health-care organizations: implications for document management	Lorence, D. P. and R. Churchill	Information Security Governance and Risk Management
Indirect effect of management support on users' compliance behaviour towards information security policies	Humaidi, N. and V. Balakrishnan	Information Security Governance and Risk Management

Title	Author	Clusters
Information accountability and usability: are there any connections?	Sahama, T., A. Kushniruk and S. Kuwata	Security Architecture and Design
Information and/or medical technology staff experience with regulations for medical information systems and medical devices	Ivarsson, B., S. Wiinberg and M. Svensson	Information Security Governance and Risk Management; Legal, Regulations, Investigations and Compliance
Information governance - a view from the NHS	Donaldson, A. and P. Walker	Information Security Governance and Risk Management
Information Security Behavior among Nurses in an Academic Hospital	Albarrak, A. I.	Information Security Governance and Risk Management
Information security concepts and practices: the case of a provincial multi-specialty hospital	Cavalli, E., A. Mattasoglio, F. Pinciroli and P. Spaggiari	Information Security Governance and Risk Management
Information Security Content Development for Awareness Training Programs in Healthcare	Ghazvini, A. and Z. Shukur	Information Security Governance and Risk Management
Information security governance: a risk assessment approach to health information systems protection	Williams, P. A.	Information Security Governance and Risk Management; Legal, Regulations, Investigations and Compliance
Information security issues that healthcare management must understand	Beaver, K.	Information Security Governance and Risk Management
Information security policies in the UK healthcare sector: a critical evaluation	Stahl, B. C., N. F. Doherty and M. Shaw	Information Security Governance and Risk Management
Information security policy's impact on reporting security incidents	Wiant, T. L.	Information Security Governance and Risk Management; Business Continuity and Disaster Recovery Planning
Information security requirements in patient-centred healthcare support systems	Alsalamah, S., W. A. Gray, J. Hilton and H. Alsalamah	Information Security Governance and Risk Management
Information security risk management for computerized health information systems in hospitals: a case study of Iran	Zarei, J. and F. Sadoughi	Information Security Governance and Risk Management
Information Security Scheme Based on Computational Temporal Ghost Imaging	Jiang, S., Y. Wang, T. Long, X. Meng, X. Yang, R. Shu and B. Sun	Cryptography
Information Security Standards for Health Information Systems: The Implementer's Approach	Kotsonis, E., S. Eliakis, A. Chryssanthou, I. Apostolakis and I. Varlamis	Information Security Governance and Risk Management
Information security: Managing the health of your laboratory information network	Klein, R. R.	Unclear
Information technologies. Physician-hospital networks	Eichenwald, S.	Unclear
Infotech. Cyber security. Health care learns to share scares and solutions	Colias, M.	Unclear
Insider threats: the myths, truths and tactics for mitigation	Brill, A. and B. Lapidus	Information Security Governance and Risk Management
Insights from nature for cybersecurity	Rzeszutko, E. and W. Mazurczyk	Security Architecture and Design
Installing an appropriate information security policy	Gaunt, N.	Information Security Governance and Risk Management
Integrating SET and EDI for secure healthcare commerce	Liu, D. R., I. C. Wu and S. T. Hsieh	Security Architecture and Design
Interference-based optical image encryption using three-dimensional phase retrieval	Chen, W. and X. Chen	Cryptography
Interpreting international governance standards for health IT use within general medical practice	Mahncke, R. J. and P. A. Williams	Information Security Governance and Risk Management
Irreversible encryption method by generation of polynomials	Quantin, C., H. Bouzelat and L. Dusserre	Cryptography
Is it safe? Security speed bumps on the information highway	Bergeron, B. P.	Information Security Governance and Risk Management; Access Control
Is patient confidentiality compromised with the electronic health record?: a position paper	Wallace, I. M.	Unclear
Is the biggest security threat to medical information simply a lack of understanding?	Williams, P. A.	Information Security Governance and Risk Management
Is your practice at risk for medical identity theft?	Weinstock, D.	Information Security Governance and Risk Management; Operations Security; Physical (Environmental) Security
It security in biomedical imaging informatics: The hidden vulnerability	Chee, W. S. A.	Operations Security; Information Security Governance and Risk Management; Business Continuity and Disaster Recovery Planning; Telecommunications and Network Security
IT security: Developing a response to increasing risks	Waegemann, C. P.	Information Security Governance and Risk Management
Job: security. 7 steps for HIPAA compliance	Johnson, L. M. and J. D. Schulte	Legal, Regulations, Investigations and Compliance; Information Security Governance and Risk Management
Lack of security of networked medical equipment in radiology	Moses, V. and I. Korah	Telecommunications and Network Security
Legal issues of the electronic dental record: security and confidentiality	Szekely, D. G., S. Milam and J. A. Khademi	Legal, Regulations, Investigations and Compliance; Information Security Governance and Risk Management
LOPD Compliance and ISO 27001 Legal Requirements in the Health Sector	Sanchez, L. E., A. S. Olmo, E. Alvarez, E. F. Medina and M. Piattini	Legal, Regulations, Investigations and Compliance

Title	Author	Clusters
m2-ABKS: Attribute-Based Multi-Keyword Search over Encrypted Personal Health Records in Multi-Owner Setting	Miao, Y., J. Ma, X. Liu, F. Wei, Z. Liu and X. A. Wang	Cryptography; Security Architecture and Design
Making Trade-Offs for Safe, Effective, and Secure Patient Care	Lyon, D.	Information Security Governance and Risk Management
Managing personal health information in distributed research network environments	Bredfeldt, C. E., A. L. Butani, R. Pardee, P. Hitz, S. Padmanabhan and G. Saylor	Information Security Governance and Risk Management
Managing secure computer systems and networks	Von Solms, B.	Telecommunications and Network Security
Managing security and privacy concerns over data storage in healthcare research	Mackenzie, I. S., B. J. Mantay, P. G. McDonnell, L. Wei and T. M. MacDonald	Information Security Governance and Risk Management
Managing the security of nursing data in the electronic health record	Samadbeik, M., Z. Gorzin, M. Khoshkam and M. Roudbari	Unclear
Medical data breaches: Notification delayed is notification denied	Kierkegaard, P.	Legal, Regulations, Investigations and Compliance
Medical image security in a HIPAA mandated PACS environment	Cao, F., H. K. Huang and X. Q. Zhou	Legal, Regulations, Investigations and Compliance; Security Architecture and Design
Medical information privacy assurance: Cryptographic and system aspects	Ateniese, G., R. Curtmola, B. de Medeiros and D. Davis	Access Control; Security Architecture and Design
Medical systems and malware	Kusche, K. P.	Telecommunications and Network Security
Method for detecting core malware sites related to biomedical information systems	Kim, D., D. Choi and J. Jin	Operations Security
Methods of responding to healthcare security incidents	Furnell, S., D. Gritzalis, S. Katsikas, K. Mavroudakis, P. Sanders and M. Warren	Information Security Governance and Risk Management; Business Continuity and Disaster Recovery Planning
mHealth data security: the need for HIPAA-compliant standardization	Luxton, D. D., R. A. Kayl and M. C. Mishkind	Software Development Security; Legal, Regulations, Investigations and Compliance
Mitigating Cybersecurity Risks	Rose, R. V. and J. S. Kass	Legal, Regulations, Investigations and Compliance
Mobile Device Security: Perspectives of Future Healthcare Workers	Hewitt, B., D. Dolezel and J. A. McLeod	Information Security Governance and Risk Management
Mobile-cloud assisted framework for selective encryption of medical images with steganography for resource-constrained devices	Sajjad, M., K. Muhammad, S. W. Baik, S. Rho, Z. Jan, S. S. Yeo and I. Mehmood	Security Architecture and Design; Cryptography
Modeling Access Control in Healthcare Organizations	Mourtou, E., A. Chryssanthou, I. Apostolakis and I. Varlamis	Access Control
Monitoring information security risks within health care	van Deursen, N., W. J. Buchanan and A. Duff	Information Security Governance and Risk Management
Multi-Level Data-Security and Data-Protection in a Distributed Search Infrastructure for Digital Medical Samples	Witt, M. and D. Krefting	Security Architecture and Design
Musings on privacy issues in health research involving disaggregate geographic data about individuals	Boulos, M. N., A. J. Curtis and P. Abdelmalik	Unclear
Network and data security design for telemedicine applications	Makris, L., N. Argiriou and M. G. Strintzis	Telecommunications and Network Security; Cryptography
Network security and data integrity in academia: an assessment and a proposal for large-scale archiving	Smith, A., D. Greenbaum, S. M. Douglas, M. Long and M. Gerstein	Telecommunications and Network Security
Network security vulnerabilities and personal privacy issues in Healthcare Information Systems: a case study in a private hospital in Turkey	Namoglu, N. and Y. Ulgen	Legal, Regulations, Investigations and Compliance; Telecommunications and Network Security
New advanced technologies to provide decentralised and secure access to medical records: case studies in oncology	Quantin, C., G. Coatrieux, F. A. Allaert, M. Fassa, K. Bourquard, J. Y. Boire, P. de Vlieger, L. Maigne and V. Breton	Access Control; Security Architecture and Design
ONLINE PUBLIC KEY CRYPTOGRAPHIC SCHEME FOR DATA SECURITY IN BRIDGE HEALTH MONITORING	Xu, F., X. Lv and R. Y. Jiang	Cryptography
Open information systems and data security in medicine	Blobel, B.	Information Security Governance and Risk Management; Access Control; Cryptography; Security Architecture and Design
Optimal information security investment in a Healthcare Information Exchange: An economic analysis	Huang, C. D., R. S. Behara and J. Goo	Information Security Governance and Risk Management
Organizational repertoires and rites in health information security	Cooper, T., J. Collmann and H. Neidermeier	Information Security Governance and Risk Management; Operations Security; Business Continuity and Disaster Recovery Planning
Organizing safety: conditions for successful information assurance programs	Collmann, J., J. Coleman, K. Sostrom and W. Wright	Information Security Governance and Risk Management; Legal, Regulations, Investigations and Compliance
Patient confidentiality in the research use of clinical medical databases	Krishna, R., K. Kelleher and E. Stahlberg	Access Control; Operations Security
Patient confidentiality, data security, and provider liabilities in diabetes management	Albisser, A. M., J. B. Albisser and L. Parker	Access Control; Cryptography; Security Architecture and Design
Patient data security in the DICOM standard	Schutze, B., M. Kroll, T. Geisbe and T. J. Filler	Security Architecture and Design
Patient privacy in the genomic era	Raisaro, J. L., E. Ayday and J. P. Hubaux	Unclear
Patients' safety, privacy and effectiveness--a conflict of interests in health care information systems?	Nymark, M.	Legal, Regulations, Investigations and Compliance; Information Security Governance and Risk Management

Title	Author	Clusters
People-centric Sensing in Assistive Healthcare: Privacy Challenges and Directions	Giannetsos, T., T. Dimitriou and N. R. Prasad	Unclear
Personal computer security - Part 1. Firewalls, antivirus software, and Internet security suites	Caruso, R. D.	Legal, Regulations, Investigations and Compliance; Access Control
Personal Data Protection in Telemedicine	Zhuravlev, M. S.	Legal, Regulations, Investigations and Compliance
Pharmaceutical digital marketing and governance: illicit actors and challenges to global patient safety and public health	Mackey, T. K. and B. A. Liang	Information Security Governance and Risk Management
Physical security, HIPPA, and the HHS wall of shame	Sage, A.	Legal, Regulations, Investigations and Compliance; Physical (Environmental) Security
Physician use of updated anti-virus software in a tertiary Nigerian hospital	Laabes, E. P., D. D. Nyango, M. M. Ayedima and N. G. Ladep	Software Development Security
Physiological Information Leakage: A New Frontier in Health Information Security	Nia, A. M., S. Sur-Kolay, A. Raghunathan and N. K. Jha	Unclear
Pitfalls in computer housekeeping by doctors and nurses in KwaZulu-Natal: no malicious intent	Jack, C., Y. Singh and M. Mars	Legal, Regulations, Investigations and Compliance; Information Security Governance and Risk Management
Policy for cryptography in healthcare--a view from the NHS	Donaldson, A.	Information Security Governance and Risk Management; Legal, Regulations, Investigations and Compliance
Policy Tech Trends 2010. Trend: privacy	Raths, D.	Legal, Regulations, Investigations and Compliance
Practical approaches to creating a security culture	Gaunt, N.	Information Security Governance and Risk Management
Practical aspects of handling data protection and data security	Louwerse, C. P.	Unclear
Practice bulletin, data security	Schraffenberger, L. A.	Unclear
Privacy and data security in E-health: requirements from the user's perspective	Wilkowska, W. and M. Ziefle	Security Architecture and Design
Privacy and information security risks in a technology platform for home-based chronic disease rehabilitation and education	Henriksen, E., T. M. Burkow, E. Johnsen and L. K. Vognild	Information Security Governance and Risk Management; Security Architecture and Design
Privacy and personal health data in cyberspace	Day, J.	Telecommunications and Network Security; Legal, Regulations, Investigations and Compliance
Privacy and Security Concerns in Healthcare Big Data: An Innovative Prescriptive	Jain, P., M. Gyanchandani and N. Khare	Security Architecture and Design
Privacy and Security in Mobile Health (mHealth) Research	Arora, S., J. Yttri and W. Nilse	Legal, Regulations, Investigations and Compliance; Security Architecture and Design
Privacy and security in the era of digital health: what should translational researchers know and do about it?	Filkins, B. L., J. Y. Kim, B. Roberts, W. Armstrong, M. A. Miller, M. L. Hultner, A. P. Castillo, J. C. Ducom, E. J. Topol and S. R. Steinhubl	Information Security Governance and Risk Management
Privacy and Security Issues Surrounding the Protection of Data Generated by Continuous Glucose Monitors	Britton, K. E. a. J. D. B.-C.	Software Development Security; Security Architecture and Design; Legal, Regulations, Investigations and Compliance
Privacy and security of patient data in the pathology laboratory	Cucoranu, I. C., A. V. Parwani, A. J. West, G. Romero-Lauro, K. Nauman, A. B. Carter, U. J. Balis, M. J. Tuthill and L. Pantanowitz	Legal, Regulations, Investigations and Compliance; Security Architecture and Design
Privacy as a Service: Protecting the Individual in Healthcare Data Processing	Su, X., J. Hyysalo, M. Rautiainen, J. Riekki, J. Sauvola, A. I. Maarala, H. Hirvonsalo, P. J. Li and H. Honko	Security Architecture and Design
Privacy Practices of Health Social Networking Sites: Implications for Privacy and Data Security in Online Cancer Communities	Charbonneau, D. H.	Information Security Governance and Risk Management
Privacy preservation and information security protection for patients' portable electronic health records	Huang, L. C., H. C. Chu, C. Y. Lien, C. H. Hsiao and T. Kao	Security Architecture and Design
Privacy-Preserving Patient-Centric Clinical Decision Support System on Naive Bayesian Classification	Liu, X., R. Lu, J. Ma, L. Chen and B. Qin	Cryptography; Security Architecture and Design
Privacy, Access Control, and Location in Mobile Applications	Zheng, Y., M. Watson, S. Chow, A. Paul, R. Bishop, R. Huang, P. C. K. Hung and J. Christie	Access Control
Privacy, confidentiality, and security in information systems of state health agencies	O'Brien, D. G. and W. A. Yasnoff	Legal, Regulations, Investigations and Compliance; Information Security Governance and Risk Management
Privacy, Confidentiality, and Security of Public Health Information	Yasnoff, W. A.	Legal, Regulations, Investigations and Compliance; Information Security Governance and Risk Management; Operations Security
Privacy, security, and the public health researcher in the era of electronic health record research	Goldstein, N. D. and A. D. Sarwate	Information Security Governance and Risk Management
Private and Efficient Query Processing on Outsourced Genomic Databases	Ghasemi, R., M. M. Al Aziz, N. Mohammed, M. H. Dehkordi and X. Jiang	Security Architecture and Design
Private and Secured Medical Data Transmission and Analysis for Wireless Sensing Healthcare System	Huang, H. P., T. H. Gong, N. Ye, R. C. Wang and Y. Dou	Telecommunications and Network Security; Security Architecture and Design; Cryptography
PROACTIVE VERSUS REACTIVE SECURITY INVESTMENTS IN THE HEALTHCARE SECTOR	Kwon, J. and M. E. Johnson	Unclear

Title	Author	Clusters
Promises and Challenges in Continuous Tracking Utilizing Amino Acids in Skin Secretions for Active Multi-Factor Biometric Authentication for Cybersecurity	Agudelo, J., V. Privman and J. Halamek	Access Control
Proposal for a security management in cloud computing for health care	Haufe, K., S. Dzombeta and K. Brandis	Information Security Governance and Risk Management
Protecting patient confidentiality in hospitals	Mulligan, E.	Legal, Regulations, Investigations and Compliance
Protection of data confidentiality and patient privacy in medical sensor networks	Sankar, R., X. H. Le, S. Lee and D. Wang	Cryptography; Security Architecture and Design
Providing integrity and authenticity in DICOM images: a novel approach	Kobayashi, L. O., S. S. Furuie and P. S. Barreto	Security Architecture and Design; Cryptography
Public Auditing with Privacy Protection in a Multi-User Model of Cloud-Assisted Body Sensor Networks	Li, S., J. Cui, H. Zhong and L. Liu	Telecommunications and Network Security
Public key infrastructures for health	Pharow, P. and B. Blobel	Security Architecture and Design; Cryptography
Quality assurance system must balance functionality with data security	Walsh, M. and F. Cortez	Security Architecture and Design
Quality optimized medical image information hiding algorithm that employs edge detection and data coding	Al-Dmour, H. and A. Al-Ani	Cryptography
Ransomware: Minimizing the Risks	Pope, J.	Legal, Regulations, Investigations and Compliance; Operations Security; Information Security Governance and Risk Management
Realizing Digital Signatures for Medical Imaging and Reporting in a PACS Environment	Lien, C. Y., T. L. Yang, C. H. Hsiao and T. Kao	Cryptography
Reducing security risk using data loss prevention technology	Beeskow, J.	Access Control; Operations Security
Releasing individually identifiable microdata with privacy protection against Stochastic threat: An application to health information	Garfinkel, R., R. Gopal and S. Thompson	Unclear
Research on medical image encryption in telemedicine systems	Dai, Y., H. Wang, Z. Zhou and Z. Jin	Cryptography
Reversible Data Hiding Based on DNA Computing	Wang, B., Y. Xie, S. Zhou, C. Zhou and X. Zheng	Cryptography
Risk analysis of information security in a mobile instant messaging and presence system for healthcare	Bones, E., P. Hasvold, E. Henriksen and T. Strandenaes	Information Security Governance and Risk Management; Security Architecture and Design
Risk and Protection of Medical Information Systems	Dimitrova, T. D.	Software Development Security; Access Control; Telecommunications and Network Security
Risk assessment of integrated electronic health records	Bjornsson, B. T., G. Sigurdardottir and S. O. Stefansson	Information Security Governance and Risk Management
Safe teleradiology: information assurance as project planning methodology	Collmann, J., A. Alaoui, D. Nguyen and D. Lindisch	Information Security Governance and Risk Management; Legal, Regulations, Investigations and Compliance
Safe: a status update on information security and the hospital community	Fundner, R.	Information Security Governance and Risk Management; Legal, Regulations, Investigations and Compliance
Safeguarding the confidentiality of automated medical information	Lawrence, L. M.	Information Security Governance and Risk Management
SAKE: scalable authenticated key exchange for mobile e-health networks	Liu, W. R., J. W. Liu, Q. H. Wu, W. Susilo, H. Deng and B. Qin	Telecommunications and Network Security; Cryptography
Secure messaging via the cloud and mobile devices: data security issues emerge with new technologies	Prestigiacomo, J.	Security Architecture and Design
Secure mobile device use in Healthcare guidance from HIP a a and ISO17799	Thomas, G. and R. A. Botha	Legal, Regulations, Investigations and Compliance
Secure privacy-preserving biometric authentication scheme for telecare medicine information systems	Li, X., Q. Wen, W. Li, H. Zhang and Z. Jin	Cryptography
Secure provision of patient-centered health information technology services in public networks-leveraging security and privacy features provided by the German nationwide health information technology infrastructure	Dehling, T. and A. Sunyaev	Security Architecture and Design
Secure public cloud platform for medical images sharing	Pan, W., G. Coatrieux, D. Bouslimi and N. Prigent	Security Architecture and Design
Secure web messaging in a pediatric chronic care clinic: a slow takeoff of kids' airmail	Hsiao, A. L., A. Bazzy-Asaad, C. Tolomeo, D. Edmonds, B. Belton and A. L. Benin	Telecommunications and Network Security
Secure Wireless Collection and Distribution of Commercial Airplane Health Data	Sampigethaya, K., R. Poovendran, L. Bushnell, M. Y. Li, R. Robinson and S. Lintelman	Security Architecture and Design; Telecommunications and Network Security
Secured ECG signal transmission for human emotional stress classification in wireless body area networks	Xu, H. S. and K. Hua	Cryptography; Telecommunications and Network Security
Securing a web-based teleradiology platform according to German law and best practices	Spitzer, M., T. Ullrich and F. Ueckert	Security Architecture and Design
Securing electronic health records with broadcast encryption schemes	Susilo, W. and K. T. Win	Cryptography; Security Architecture and Design
Securing electronic medical records transmissions over unsecured communications: An overview for better medical governance	Alanazi, H. O., H. A. Jalab, G. M. Alam, B. B. Zaidan and A. A. Zaidan	Security Architecture and Design; Cryptography

Title	Author	Clusters
SECURING M-HEALTHCARE SOCIAL NETWORKS: CHALLENGES, COUNTERMEASURES AND FUTURE DIRECTIONS	Zhou, J., Z. F. Cao, X. L. Dong, X. D. Lin and A. V. Vasilakos	Security Architecture and Design
Securing medical research: a cybersecurity point of view	Schneier, B.	Cryptography
Securing Patient Data in Wireless Body Area Sensor Network Using Biometrics Based Key Generation	Geetha, K., S. Chitra, B. Madhusudhanan and X. Z. Gao	Cryptography
Securing SSL-VPN with LR-AKE to access personal health record	Eizen, K., S. Masato, K. Kazukuni, N. Yoshihito, K. Takuji and I. Ken	Cryptography; Security Architecture and Design
Security Analysis of Standards-Driven Communication Protocols for Healthcare Scenarios	Masi, M., R. Pugliese and F. Tiezzi	Security Architecture and Design; Information Security Governance and Risk Management
Security and access of health research data	Susilo, W. and K. T. Win	Cryptography
Security and confidentiality in an electronic medical record	Olson, L. A., S. G. Peters and J. B. Stewart	Information Security Governance and Risk Management; Operations Security
Security and privacy qualities of medical devices: an analysis of FDA postmarket surveillance	Kramer, D. B., M. Baker, B. Ransford, A. Molina-Markham, Q. Stewart, K. Fu and M. R. Reynolds	Software Development Security
Security and privacy requirements for a multi-institutional cancer research data grid: an interview-based study	Manion, F. J., R. J. Robbins, W. A. Weems and R. S. Crowley	Information Security Governance and Risk Management; Legal, Regulations, Investigations and Compliance
Security aspects of medical file regrouping for the epidemiological follow-up	Quantin, C., E. Kerkri, F. A. Allaert, H. Bouzelat and L. Dusserre	Security Architecture and Design
Security aspects of teleradiology between the university centre and outlying hospitals in Tyrol	Sogner, P., K. Goidinger, D. Reiter, A. Stoeger and D. z. Nedden	Access Control
Security Attacks and Solutions in Electronic Health (E-health) Systems	Zeadally, S., J. T. Isaac and Z. Baig	Security Architecture and Design; Cryptography
Security Audit Center--a suggested model for effective audit strategies in health care informatics	Hayam, A.	Information Security Governance and Risk Management; Operations Security
Security breaches: tips for assessing and limiting your risks	Coons, L. R.	Information Security Governance and Risk Management; Access Control; Business Continuity and Disaster Recovery Planning
Security challenges in integration of a PHR-S into a standards based national EHR	Mense, A., F. Hoheiser Pfortner and S. Sauermann	Security Architecture and Design
Security considerations for e-mental health interventions	Bennett, K., A. J. Bennett and K. M. Griffiths	Security Architecture and Design; Information Security Governance and Risk Management; Access Control; Physical (Environmental) Security
Security development of a pocket-sized teleradiology consultation system	Niinimaki, J., A. Holopainen, J. Kerttula and J. Reponen	Security Architecture and Design
Security in health-care information systems--current trends	Smith, E. a. J. H. E.	Access Control; Information Security Governance and Risk Management
Security in the Internet	Seibel, R. M. M., K. Kocher and P. Landsberg	Telecommunications and Network Security; Cryptography
Security of electronic medical information and patient privacy: what you need to know	Andriole, K. P.	Information Security Governance and Risk Management; Security Architecture and Design
Security of electronic mental health communication and record-keeping in the digital age	Elhai, J. D. and B. C. Frueh	Information Security Governance and Risk Management
Security of healthcare data networks used for epidemiological studies	Quantin, C., F. A. Allaert, H. Bouzelat, J. M. Rodrigues, B. Trombert-Paviot, P. Brunet-Lecomte, F. Gremy and L. Dusserre	Software Development Security
Security of healthcare information systems based on the CORBA middleware	Blobel, B. and M. Holena	Security Architecture and Design; Information Security Governance and Risk Management
Security of patient data when decommissioning ultrasound systems	Moggridge, J.	Software Development Security
Security of the distributed electronic patient record: a case-based approach to identifying policy issues	Anderson, J. G.	Information Security Governance and Risk Management
Security of the electronic health care record--professional and ethical implications	Gaunt, N. and F. Roger-France	Information Security Governance and Risk Management
Security practices and regulatory compliance in the healthcare industry	Kwon, J. and M. E. Johnson	Information Security Governance and Risk Management; Legal, Regulations, Investigations and Compliance
Security requirements and solutions in electronic health records: lessons learned from a comparative study	Farzandipour, M., F. Sadoughi, M. Ahmadi and I. Karimi	Access Control; Software Development Security; Operations Security
Security requirements for electronic patients records: the Norwegian view	Iversen, K. R.	Unclear
Security standards for medical information systems	Humphreys, T.	Legal, Regulations, Investigations and Compliance; Information Security Governance and Risk Management
Security threats and solutions in distributed, interoperable health information systems using middleware	Blobel, B. and M. Holena	Security Architecture and Design; Information Security Governance and Risk Management
Security threats and trends in society	Roger, F. H.	Unclear
Security threats are usually an inside job	Simpson, R. L.	Information Security Governance and Risk Management
Security threats categories in healthcare information systems	Samy, G. N., R. Ahmad and Z. Ismail	Information Security Governance and Risk Management; Operations Security

Title	Author	Clusters
Security Tradeoffs in Cyber Physical Systems: A Case Study Survey on Implantable Medical Devices	Altawy, H. and A. M. Youssef	Access Control
Security, privacy, and confidentiality issues on the Internet	Kelly, G. and B. McKenzie	Telecommunications and Network Security
Security: keeping the flame alive	Amatayakul, M.	Legal, Regulations, Investigations and Compliance; Information Security Governance and Risk Management
Serious technology assessment for health care information technology	Cushman, R.	Unclear
Seven layers of security to help protect biomedical research facilities	Mortell, N.	Operations Security
Shared Electronic Health Record Systems: Key Legal and Security Challenges	Christiansen, E. K., E. Skipenes, M. F. Hausken, S. Skeie, T. Ostbye and M. M. Iversen	Access Control; Legal, Regulations, Investigations and Compliance; Information Security Governance and Risk Management
Sharing with Care An Information Accountability Perspective	Gajanayake, R., R. Iannella and T. Sahama	Unclear
Shortcomings of current grid middlewares regarding privacy in HealthGrids	Mohammed, Y., U. Sax, F. Viezens and O. Rienhoff	Security Architecture and Design
Silicon photonic physical unclonable function	Grubel, B. C., B. T. Bosworth, M. R. Kossey, H. Sun, A. B. Cooper, M. A. Foster and A. C. Foster	Security Architecture and Design
Six opinions on IT security. Panel discussion	Fieldhouse, D., J. Lanson, K. MacDonald, A. Shar, K. Stanfield and C. Zak	Information Security Governance and Risk Management; Legal, Regulations, Investigations and Compliance
Smartphone and mobile phone security for the clinician	Barber, H.	Unclear
SPOC: A Secure and Privacy-Preserving Opportunistic Computing Framework for Mobile-Healthcare Emergency	Lu, R. X., X. D. Lin and X. M. Shen	Access Control; Security Architecture and Design
Standards for confidentiality, privacy, access, and data security	Hanken, M. A.	Information Security Governance and Risk Management
Statistical Models for EHR Security in Web Healthcare Information Systems	Zimeras, S., A. N. Kastania, A. Chryssanthou, I. Apostolakis and I. Varlamis	Security Architecture and Design
Steganography and encrypting based on immunochemical systems	Kim, K. W., V. Bocharova, J. Halamek, M. K. Oh and E. Katz	Cryptography
Strategic approach to information security and assurance in health research	Akazawa, S., M. Igarashi, H. Sawa and H. Tamashiro	Information Security Governance and Risk Management; Legal, Regulations, Investigations and Compliance; Security Architecture and Design
Synthetic hardware performance analysis in virtualized cloud environment for healthcare organization	Tan, C. H. and Y. W. Teh	Unclear
Taiwan's perspective on electronic medical records' security and privacy protection: lessons learned from HIPAA	Yang, C. M., H. C. Lin, P. Chang and W. S. Jian	Legal, Regulations, Investigations and Compliance
Taking aim at medical identity theft. Document security key element to comply with government regulations	Raymond, C.	Legal, Regulations, Investigations and Compliance
Teaching EHRs security with simulation for non-technical healthcare professionals	Gaynor, M., T. Omer and J. S. Turner	Information Security Governance and Risk Management
Telemedicine in healthcare. 2: The legal and ethical aspects of using new technology	Sarhan, F.	Legal, Regulations, Investigations and Compliance
The A to Z of healthcare data breaches	Kobus, T. J., 3rd	Information Security Governance and Risk Management; Physical (Environmental) Security
The Australian PCEHR System: Ensuring Privacy and Security through an Improved Access Control Mechanism	Vimalachandran, P., H. Wang, Y. Zhang and G. Zhuo	Access Control
The cost of IT security	McMillan, M.	Unclear
The effects of different representations on static structure analysis of computer malware signatures	Narayanan, A., Y. Chen, S. Pang and B. Tao	Cryptography
The Electronic Wardeni management of the data security access in a heterogeneous university hospital environment in Belgium	Piret, C., F. H. France, D. Clae and F. Dubr	Software Development Security
The enhancement of security in healthcare information systems	Liu, C. H., Y. F. Chung, T. S. Chen and S. D. Wang	Telecommunications and Network Security; Security Architecture and Design
The health information system security threat lifecycle: An informatics theory	Fernando, J. I. and L. L. Dawson	Information Security Governance and Risk Management
The HIPAA Security Rule: implications for biomedical devices		Legal, Regulations, Investigations and Compliance
The Impact of the Security Competency on Self-Efficacy in Information Security for Effective Health Information Security in Iran	Shahri, A. B., Z. Ismail and S. Mohanna	Unclear
The information security needs in radiological information systems-an insight on state hospitals of Iran, 2012	Farhadi, A. and M. Ahmadi	Access Control
The legal and ethical aspects of telemedicine. 2: Data protection, security and European law	Stanberry, B.	Legal, Regulations, Investigations and Compliance

Title	Author	Clusters
The Medical Science DMZ	Peisert, S., W. Barnett, E. Dart, J. Cuff, R. L. Grossman, E. Balas, A. Berman, A. Shankar and B. Tierney	Security Architecture and Design; Telecommunications and Network Security
The Micro, Meso, and Macro Perspectives of HIT Adoption	Sridhar, D. S. and D. S. Sridhar	Legal, Regulations, Investigations and Compliance
THE MODERATING EFFECT OF WORKING EXPERIENCE ON HEALTH INFORMATION SYSTEM SECURITY POLICIES COMPLIANCE BEHAVIOUR	Humaidi, N. and V. Balakrishnan	Information Security Governance and Risk Management
The Natural Hospital Environment: a Socio-Technical-Material perspective	Fernando, J. a. L. D.	Information Security Governance and Risk Management
The network and its role in digital imaging and communications in medicine imaging	Ballance, D.	Telecommunications and Network Security
The new confidentiality for the 21st century in a managed care environment	Rock, B. and E. Congress	Security Architecture and Design
The organisation and management of information security issues in health care	Louwerse, C. P.	Unclear
The patient register	Antony, G., K. Eggert, S. Franke and W. H. Oertel	Telecommunications and Network Security; Security Architecture and Design
The Rise of Ransomware	Berlin, J.	Business Continuity and Disaster Recovery Planning; Operations Security; Information Security Governance and Risk Management
The role of information security learning and individual factors in disclosing patients' health information	Park, E. H., J. Kim and Y. S. Park	Information Security Governance and Risk Management
The role of physical security in the information security world	Carey, J.	Physical (Environmental) Security
The role of privacy protection in healthcare information systems adoption	Hsu, C. L., M. R. Lee and C. H. Su	Information Security Governance and Risk Management; Access Control
The Secure Medical Research Workspace: An IT Infrastructure to Enable Secure Research on Clinical Data	Shoffner, M., P. Owen, J. Mostafa, B. Lamm, X. S. Wang, C. P. Schmitt and S. C. Ahalt	Security Architecture and Design
The security of patient identifiable information in doctors' homes	McLean, I. and C. M. Anderson	Information Security Governance and Risk Management
Think Like a Hacker	Khera, M.	Software Development Security; Telecommunications and Network Security
THREATS AND SOLUTIONS FOR THE SECURITY OF ELECTRONIC PATIENT RECORD (EPR) IN A DEVELOPING COUNTRY	Kahouei, M., J. M. Zadeh and Z. Abbasi	Information Security Governance and Risk Management
Threats to information security of real-time disease surveillance systems	Henriksen, E., M. A. Johansen, A. Baardsgaard and J. G. Bellika	Information Security Governance and Risk Management
Toward Proper Authentication Methods in Electronic Medical Record Access Compliant to HIPAA and C.I.A. Triangle	Tipton, S. J., S. Forkey and Y. B. Choi	Legal, Regulations, Investigations and Compliance; Access Control
Toward secure distribution of electronic health records: quantitative feasibility study on secure E-mail systems for sharing patient records	Gomi, Y., H. Nogawa and H. Tanaka	Cryptography; Information Security Governance and Risk Management
Towards secure Grid-enabled healthcare	Power, D. J., E. A. Politou, M. A. Slaymaker and A. C. Simpson	Security Architecture and Design
Transforming Scientific Inquiry: Tapping Into Digital Data by Building a Culture of Transparency and Consent	Smith, R. J., D. Grande and R. M. Merchant	Unclear
Trusted third party services for deploying secure telemedical applications over the WWW	Spinellis, D., S. Gritzalis, J. Iliadis, D. Gritzalis and S. Katsikas	Security Architecture and Design; Cryptography
UK National Data Guardian for Health and Care's Review of Data Security: Trust, better security and opt-outs	Chan, T., C. T. Di Iorio, S. De Lusignan, D. Lo Russo, C. Kuziemsky and S. T. Liaw	Legal, Regulations, Investigations and Compliance
Uniting security forces against risk	Wagner, L.	Unclear
Use of a secure Internet Web site for collaborative medical research	Marshall, W. W. and R. W. Haley	Information Security Governance and Risk Management; Telecommunications and Network Security
Use of the ISO/IEC 17799 framework in healthcare information security management	Posthumus, L.	Legal, Regulations, Investigations and Compliance; Information Security Governance and Risk Management
USING DIGITAL SIGNATURES IN WIRELESS PORTABLE REMOTE HEALTH MONITORING SYSTEMS	Aslantas, V., R. Kurban and T. Caglikantar	Cryptography
Using Fingerprint Features as Personalized Cryptographic key in Personal Health Record Systems	Ma, G. Q., J. Liu and B. Ni	Cryptography
Value conflicts for information security management	Hedstrom, K., E. Kolkowska, F. Karlsson and J. P. Allen	Information Security Governance and Risk Management
Video calls from lay bystanders to dispatch centers - risk assessment of information security	Bolle, S. R., P. Hasvold and E. Henriksen	Information Security Governance and Risk Management
Visual sharing protection method for medical images	Chen, W. K. and H. K. Tso	Cryptography
Vulnerability to chosen-plaintext attack of optoelectronic information encryption with phase-shifting interferometry	Qin, W., X. Peng, X. Meng and B. Z. Gao	Cryptography

Title	Author	Clusters
Watermarking - a new way to bring evidence in case of telemedicine litigation	Coatrieux, G., C. Quantin, F. A. Allaert, B. Auverlot and C. Roux	Legal, Regulations, Investigations and Compliance; Security Architecture and Design
What caused the breach? An examination of use of information technology and health data breaches	Wikina, S. B.	Physical (Environmental) Security; Operations Security; Information Security Governance and Risk Management
What strategies can help us sensibly manage patient information?	Skuteris, L. R.	Information Security Governance and Risk Management
What to do before disaster strikes	Simpson, R. L.	Business Continuity and Disaster Recovery Planning
When Do IT Security Investments Matter? Accounting for the Influence of Institutional Factors in the Context of Healthcare Data Breaches	Angst, C. M., E. S. Block, J. D'Arcy and K. Kelley	Information Security Governance and Risk Management; Operations Security
When it comes to securing patient health information from breaches, your best medicine is a dose of prevention: A cybersecurity risk assessment checklist	Blanke, S. J. and E. McGrady	Unclear
When trust defies common security sense	Williams, P. A.	Information Security Governance and Risk Management
WHO'S PHISHING FOR YOUR DATA?	Aston, G.	Information Security Governance and Risk Management; Operations Security; Access Control
Why information security belongs on the CFO's agenda	Quinnild, J., J. Fusile and C. Smith	Information Security Governance and Risk Management
WOC practice in cyberspace: legal and ethical issues	Hoyman, K.	Legal, Regulations, Investigations and Compliance; Information Security Governance and Risk Management
Workarounds to computer access in healthcare organizations: you want my password or a dead patient?	Koppel, R., S. Smith, J. Blythe and V. Kothari	Information Security Governance and Risk Management
Zip it!	Conde, C.	Legal, Regulations, Investigations and Compliance

* Articles that could not fit into any of the ten clusters were marked as “unclear.”

S3. Supplementary Figures

Figure S1 includes the publication line as well as the number of authors per year. The gap between the number of authors and the number of articles per year has continued to increase as the average ratio of authors to articles has risen. Articles with multiple authors are increasingly being published.

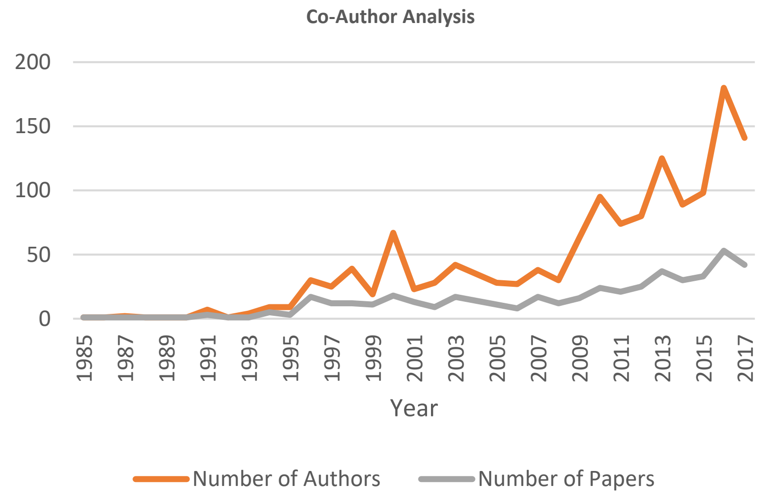

Figure S1: Co-author analysis

The majority of the articles that had multiple high-level interdisciplinary categorizations were technological-managerial (42.1%), and managerial-legal (28.4%) and legal-technological (23.1%) were close behind. Additionally, six out of the 95 interdisciplinary articles (6.3%) had all three managerial-legal-technological clusters.

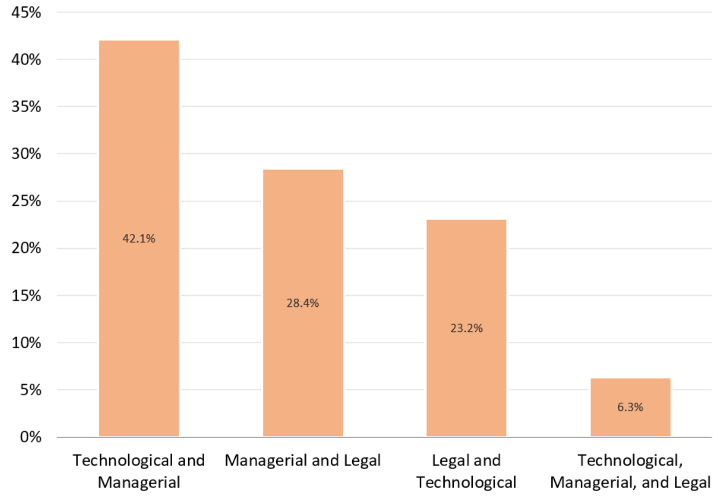

Figure S2: Interdisciplinary cluster details

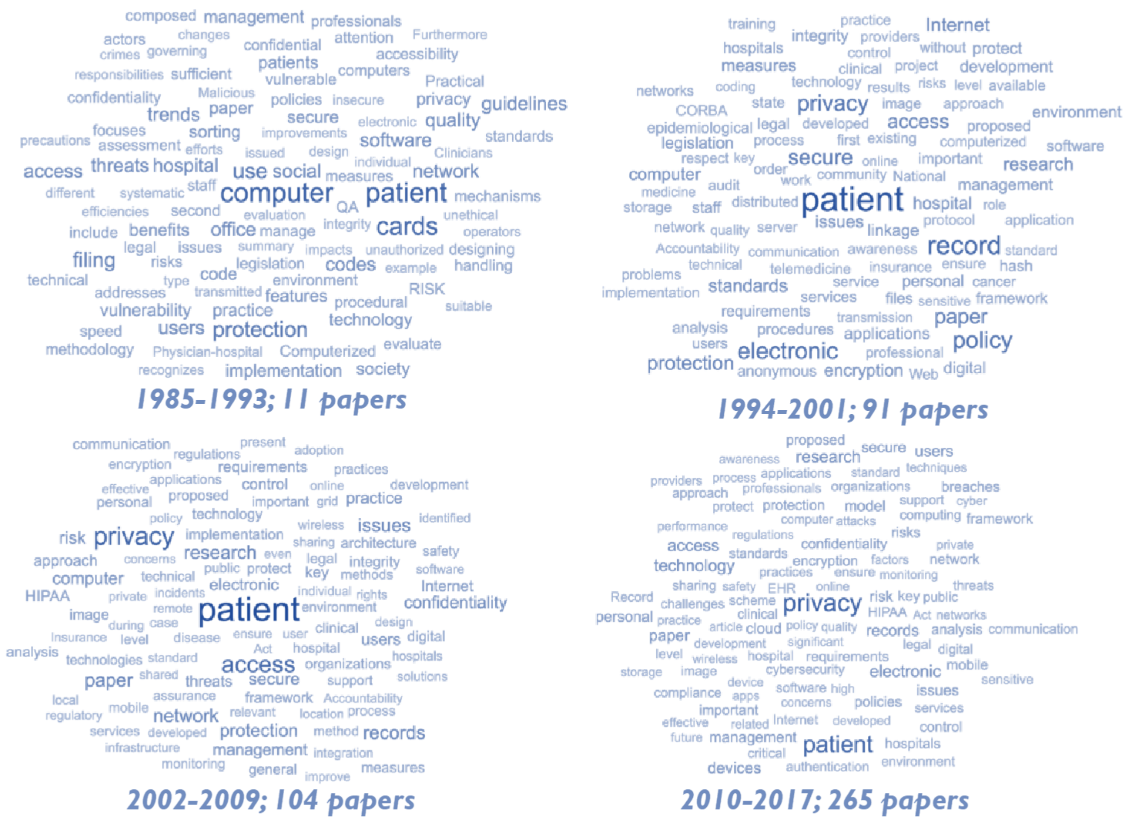

Figure S3: Word clouds of titles and abstracts of articles in four time periods

Analyzing Figures S4 through S7, we begin to see “policies” and “analysis” and ideas related to “policies,” such as “HIPAA” and “legal,” in the period beginning in 1994. From 1985 to 2009, many articles focus on the security and management of systems. Starting in 1994, the idea of “protection” starts to emerge, gradually increasing its presence and range, and including words like “confidentiality” and “breaches.” In the map for the period 1985 to 1993, there is little overlap between bubbles, and the distance between exact words (the grey dots) are also larger.

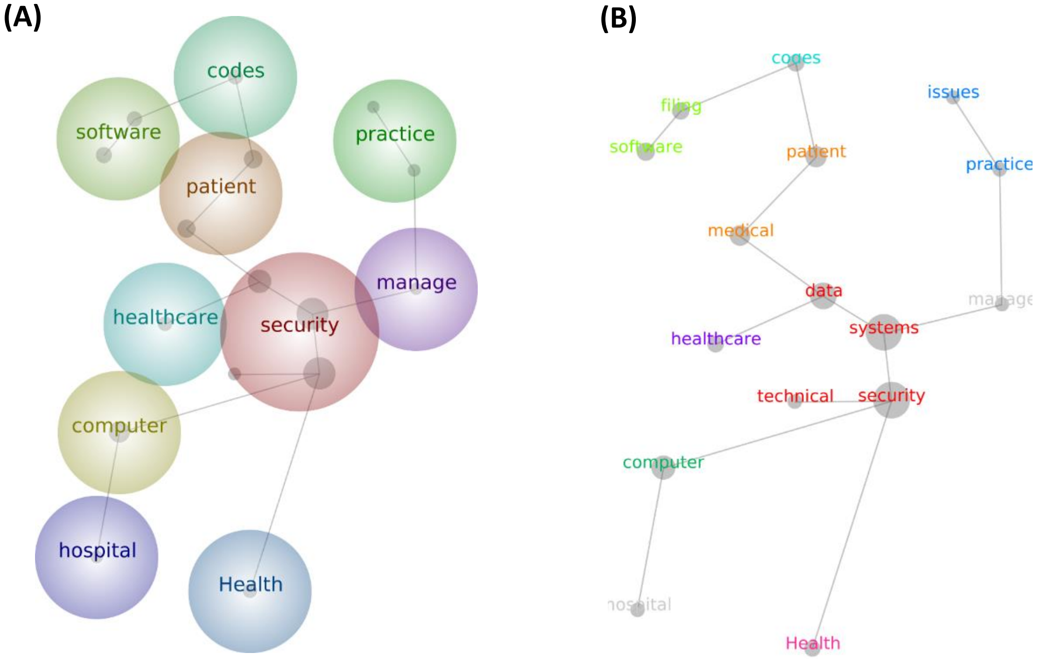

Figure S4: Thematic map 1985-1993 (A) and concept cloud 1985-1993 (B)

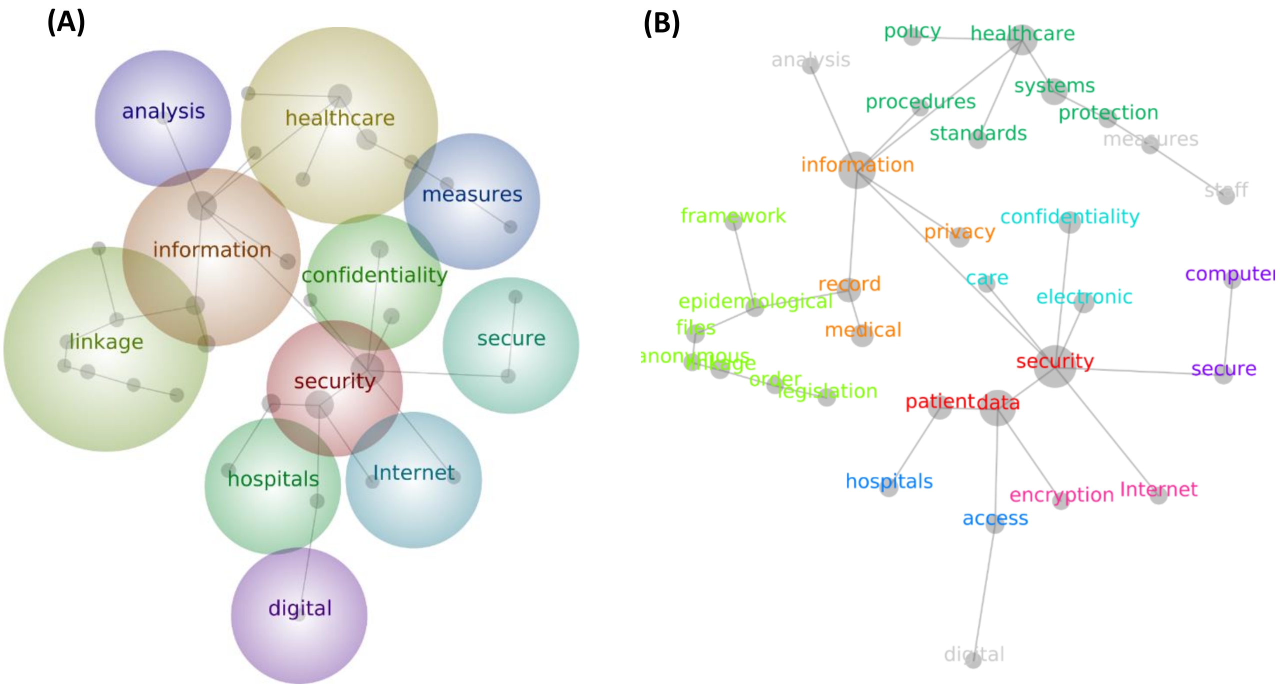

Figure S5: Thematic map 1994-2001 (A) and concept cloud 1994-2001 (B)

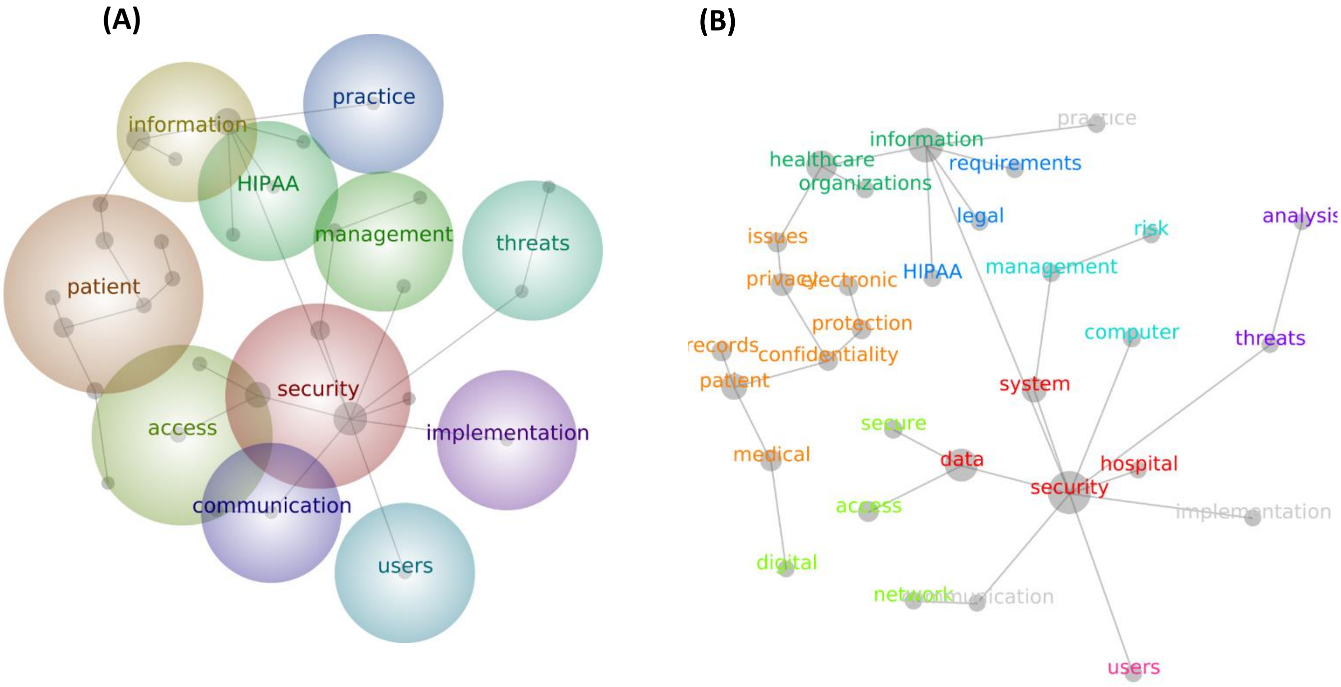

Figure S6: Thematic map 2002-2009 (A) and concept cloud 2002-2009 (B)

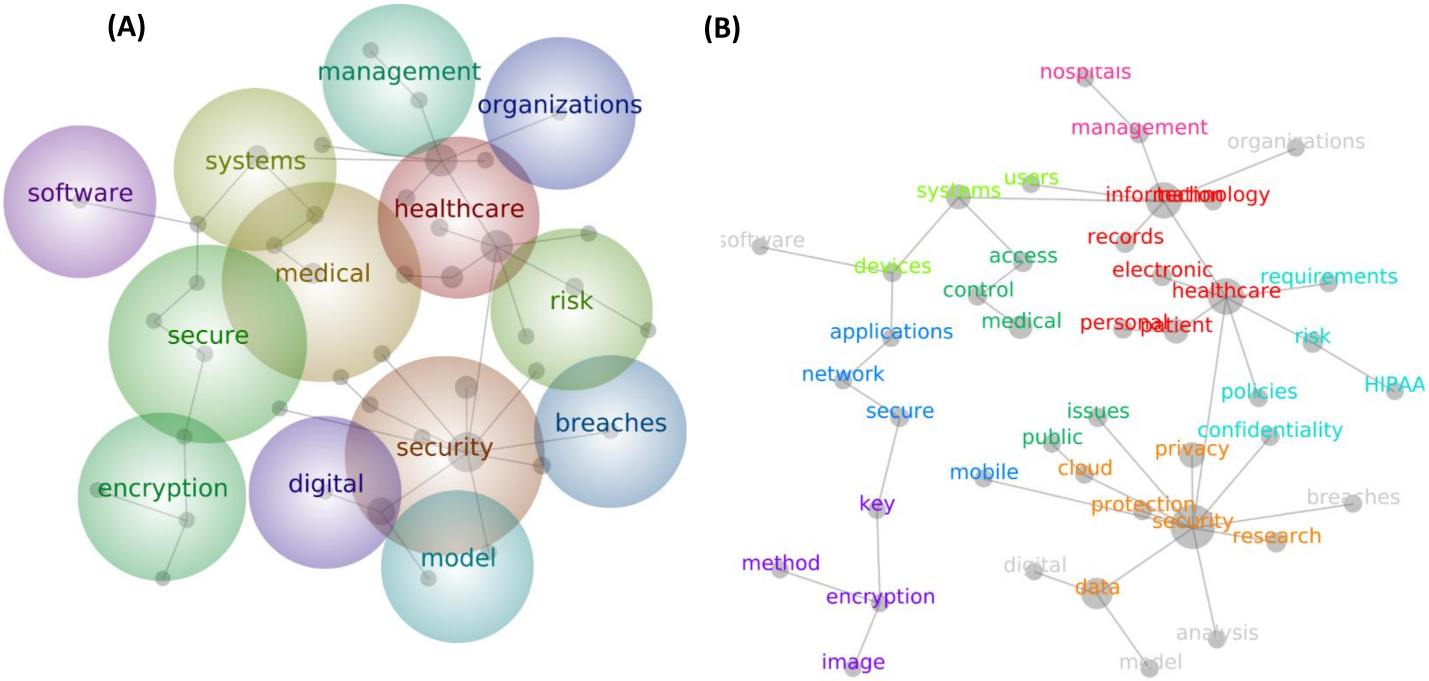

Figure S7: Thematic map 2010-2017 (A) and concept cloud 2010-2017(B)
